# Spike-Threshold Variability Originated from Separatrix-Crossing in Neuronal Dynamics

**DOI:** 10.1038/srep31719

**Published:** 2016-08-22

**Authors:** Longfei Wang, Hengtong Wang, Lianchun Yu, Yong Chen

**Affiliations:** 1 Institute of Theoretical Physics, Lanzhou University, Lanzhou, Gansu 730000, China; 2 College of Physics and Information Technology, Shaanxi Normal University, Xi’an 710062, China; 3 Center of Soft Matter Physics and its Application, Beihang University, Beijing 100191, China; 4 School of Physics and Nuclear Energy Engineering, Beihang University, Beijing 100191, China

## Abstract

The threshold voltage for action potential generation is a key regulator of neuronal signal processing, yet the mechanism of its dynamic variation is still not well described. In this paper, we propose that threshold phenomena can be classified as parameter thresholds and state thresholds. Voltage thresholds which belong to the state threshold are determined by the ‘general separatrix’ in state space. We demonstrate that the separatrix generally exists in the state space of neuron models. The general form of separatrix was assumed as the function of both states and stimuli and the previously assumed threshold evolving equation versus time is naturally deduced from the separatrix. In terms of neuronal dynamics, the threshold voltage variation, which is affected by different stimuli, is determined by crossing the separatrix at different points in state space. We suggest that the separatrix-crossing mechanism in state space is the intrinsic dynamic mechanism for threshold voltages and post-stimulus threshold phenomena. These proposals are also systematically verified in example models, three of which have analytic separatrices and one is the classic Hodgkin-Huxley model. The separatrix-crossing framework provides an overview of the neuronal threshold and will facilitate understanding of the nature of threshold variability.

Neurons encode stimuli into time sequences of stereotypical membrane potential pulses that are known as action potentials (APs). The firing of an AP is thought to be determined by whether the membrane potential exceeds a certain threshold value; however, the threshold (following the routine in neuroscience, ‘threshold’ in this paper means ‘threshold voltage/membrane potential’ unless particular threshold types are specified) is not a constant value. Threshold variation (i.e., dynamic thresholds) has been observed in the electrophysiological experiments both *in vivo*^1^,^2^,^3^,^4^,^5^,^6^,^7^,^8^ and *in vitro*^6^,^9^,^10^. Dynamic thresholds can shape the responses of neurons^6^ and enhance coincidence detection^2^,^3^,^5^ as well as improve the feature selectivity^8^ of single neurons, while filter weak asynchronous activity in neuronal networks^3^,^11^. Threshold variability also participates in precise temporal coding^9^,^12^,^13^ and influences metabolic energy efficiency^14^.

Although the dynamic threshold plays an important role in neuronal information processing, its mechanism has not been well characterized. A pioneering study of threshold “accommodation” assumed that the threshold voltage follows a simple temporal exponential process^15^. Accordingly, similar first-order kinetic equations that describe threshold variation are still used by contemporary researchers^6^,^9^,^16^,^17^,^18^,^19^. The equation has the following form:


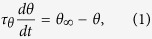


where *θ* denotes the dynamic threshold, *θ*_∞_ is a constant threshold and *τ*_*θ*_ is the time constant. In investigating of the quantitative laws of AP generation, Hodgkin and Huxley found that the threshold could be increased by *Na*^+^ channel inactivation and *K*^+^ channel activation and suggested that the threshold might be a function of the membrane potential^20^,^21^. Indeed, recent experiments have shown that the threshold of cortical neurons *in vivo* positively correlates with the membrane potential and negatively correlates with the rate of membrane depolarization^1^,^2^,^3^. Additionally, more ion channel types may participate in determining the threshold variation^22^,^23^. In principle, the relationship of threshold with either the membrane potential or the rising rate is determined by the activation and inactivation of all ion channels^24^,^25^.

Although the biophysical mechanism has not been well studied by neuroscientists, mathematical classifications and mechanisms describing the neuronal threshold, especially the separatrix concept and quasi-threshold phenomena, were proposed by FitzHugh in 1950s^26^. However, these mechanisms have not captured the attention of scientists in a long time, until recently, when similar concepts have been hypothesized or reintroduced by researchers^19^,^27^,^28^. Several experimental works on the threshold variation adopted similar methods or obtained results that were derived from these concepts; threshold was defined as the threshold voltage value at the time point that a stimulus is switched off^9^,^24^,^28^, and simple dynamic threshold equations similar to equation (1) were adopted^6^,^9^,^18^. Additionally, Platkiewicz and Brette deduced a simplified threshold equation that could quantify the contribution of different ion channels and synaptic conductances to spike thresholds^16^,^17^, and a similar form of equation (1) is also derivable from their equations^16^. However, the general mechanism of dynamic threshold for AP generation in a specified neuron has not been well described, especially when the neuron is subjected to differing stimuli, such as a rectangular pulse or a ramp current^9^,^24^,^28^. To connect the theory and experiment results, and considering the recent advances on the threshold phenomena associated with large difference between the time scales of fast and slow variables^29^,^30^ that explain the quasi-threshold well^26^, we introduce the general notion of *separatrix*, which is a boundary separating two different modes of dynamic behavior in state space. The general separatrix is equivalent to the concept of ‘threshold manifold’ in mathematics^31^,^32^,^33^ and the concept of ‘switching manifold’ in control sciences^19^. Two different mechanisms exist that describe the threshold voltages in two different types of neuronal models: one is the separatrix of fixed points^26^,^31^ and the other is the quasi-separatrix or canard^26^,^29^,^31^,^32^. The general separatrix is able to incorporate both the ‘real-separatrix’ (rigid mathematical separatrix, the manifolds of fixed points^26^) and the ‘quasi-separatrix’^29^,^32^,^34^ (‘ghost separatrix’ in refs 35, 36, 37). The general separatrix can provide an adequate definition for threshold voltage and a uniform mechanism to explain numerous threshold phenomena.

In the current study, we first propose that the threshold phenomena in excitable neurons can be classified as ‘parameter threshold’ and ‘state threshold’. We postulate that threshold voltages belong to the state threshold and have a mechanism distinguished from the bifurcation theory that explains parameter threshold. We demonstrate that the general separatrix exists in the full state space of different types of excitable neurons. Crossing the separatrix, i.e., *separatrix-crossing*, at different sites causes state threshold phenomena and threshold variation. Then, we introduce a general form of a separatrix in a common excitable conductance-based neuron model. According to the form of a separatrix, the general threshold evolving equation versus time is deduced and transformed into equation (1) for the simplest case.

Using the separatrix-crossing framework, we then analyze the threshold voltages and threshold phenomena in different models. The threshold set (i.e., separatrices) is not a fixed value but is a varying threshold point in a Quadratic Integrate-and-Fire (QIF) model^31^, a set of threshold curves in a two-dimensional (2D) model, a threshold plane in a three-dimensional (3D) piecewise linear (PWL) model and a threshold hypersurface in the Hodgkin-Huxley model. Additionally, the threshold sets of the aforementioned models dynamically vary with external stimuli. Through the simple but analytic threshold function in the QIF model as well as in the 2D and 3D PWL model, we demonstrate that separatrix-crossing in the state space is one origin of threshold variability. We also deduce the threshold variation equations in these models and determine whether the equations can be simplified to equation (1). State dependent threshold parameters, e.g., the threshold timing and amplitude of the voltage clamp, step current and ramp current, are also determined by separatrix in these models. Additionally, the numerical simulation of threshold phenomena in the Hodgkin-Huxley model following voltage clamping demonstrates that the separatrix-determined threshold is valid in actual neurons that exhibit complex dynamics.

## Results

### Classification and mechanisms of threshold phenomena

Neuronal threshold phenomena can be classified into different types. In general, neuronal threshold phenomena can be categorized by their application domains, which are as follows: *in vitro*, *in vivo* and *in model*^16^. Threshold phenomena in neural models have been mathematically divided into *discontinuous*-, *singular-point*- and *quasi threshold phenomena* (DTP, STP and QTP in abbreviation) by FitzHugh^26^. In the current study, we propose that the threshold can be generally categorized as either the *parameter threshold* or the *state threshold*. The parameter threshold depends on bifurcation while the state threshold is determined by the initial- or boundary value problem. The two common neuronal threshold phenomena, the threshold voltage and threshold current (usually the amplitude of direct current, i.e., DC), belong to the state threshold and parameter threshold, respectively.

The classification is suitable for both theoretical use and practical application. “Parameter” and “state” have distinct meanings in models: the parameters will not change, and the states are variables. In reality, a piece of functional nerve membrane have several intrinsic statistic properties, for example, the membrane potential, the states of different subunits of ion channels, the number of different ion channels available and the reversal potentials for different ion channel types. The former two quantities are time-varying, so we call them ‘states’ (or ‘variable’ in the model), while the latter two do not change, so these properties are treated as parameters. The biophysical properties in reality have exact correspondents in the conductance-based model. Taking the Hodgkin-Huxley model as an example: variables *V*, *m*, *h* and *n* form a 4-dimensional state space, while the maximum conductance of ion channels 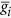 (*i* = *Na*, *K*, *L*) and reversal potential *E*_*i*_ (*i* = *Na*, *K*, *L*) are all parameters.

In electrophysiological experiments, besides of the intrinsic factors, current can be artificially injected by external circuits or received from synapses of upstream neurons. The external current is an independent variable and was treated as a state variable by FitzHugh^26^. However, the amplitude of DC or step current is unique and can be recognized as a parameter because the current is time-invariant as the parameters do. According to the bifurcation theory, the current amplitude is the most common bifurcation parameter. The varying current with special fixed forms, such as periodic or pulse current, may involve parameters such as amplitude, duration and period. These quantities may be called ‘parameters’ for the current, but the current varies in terms of time. Therefore, the threshold phenomena for these quantities is hybrid.

Different types of threshold phenomena have different mechanisms. The parameter threshold is usually explained by bifurcation theory, e.g., threshold amplitude of DC, while the mechanism of the state threshold is related to the boundary separating dynamic behaviors–separatrix. In the current study, we demonstrate that the separatrix determines time-varying threshold phenomena, including the threshold voltages and state-dependent threshold quantities, e.g., threshold amplitude and time of step or ramp current.

The classification of parameters and state is rigid in principle; however, considering both the significance and time scale of variables at the threshold, some approximations can be made. A variable with small significance in threshold variation can be approximated as a parameter. The quasi-static effect with large significance should be treated carefully; for example, the synaptic current with a long decay may greatly change the behavior of a neuron by slowly varying the threshold in a long time scale, which can not be explained otherwise^33^. These approximations are considered in the modeling process. Once the model is established, the classification of parameters and states is clear.

### A general mechanism of threshold voltage and threshold variation

We begin with the general equations of an excitable neuron. Since the AP generation threshold depends on the complex interaction of different ion channels and the stimulus^22^,^23^,^24^, it is necessary to distinguish gating variables, membrane potential and external current. On the other hand, considering the important role of adaptation of the ion channels in threshold variation, we directly write the time derivative of ion channel gating variables in the the adaptation form. In contrast to the more general differential equations in mathematics^26^,^38^, we construct the general equations of a single compartment excitable conductance-based neuron as


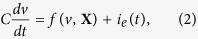



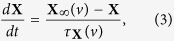


where *v* is the membrane potential and **X** is the vector of the gating variables of the ion channels; they are abbreviations of *v*(*t*) and **X(t)**, respectively. *i*_*e*_(*t*) is the external current stimulus and *f*(*v*, **X**) represents the intrinsic ion currents. The equations allow us to focus on the dynamic threshold analysis and assist in deriving the first-order threshold equation.

**Figure 1:**
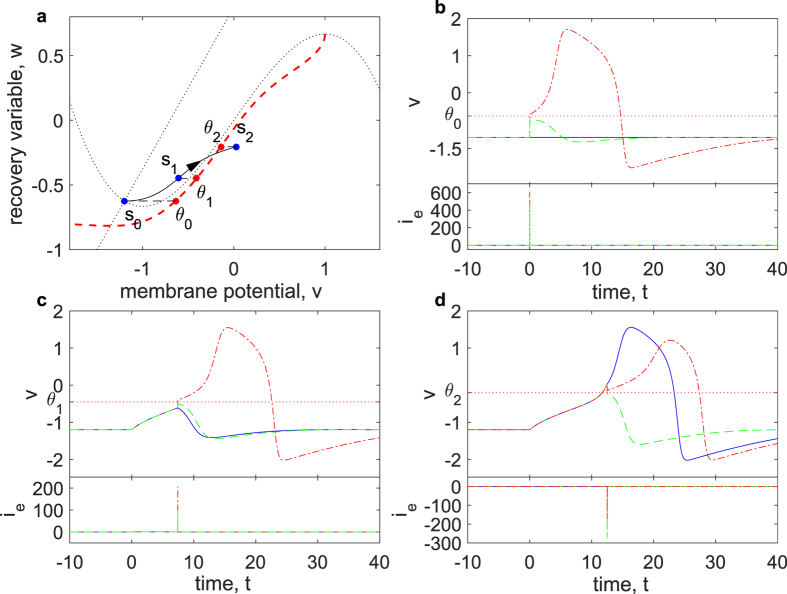
Instantaneous threshold is defined by whether an AP will be induced when only varying the membrane potential. (**a**) Threshold voltages are the minimum/maximum values that determine AP initiation when shifting the membrane potential only in state space. Shifting state point *s*_*i*_ (in this figure caption, *i* = 0, 1 or 2) in the *v*-axis will produce the threshold *θ*_*i*_ in the separatrix (red dashed line). State *s*_0_ is the resting state, and the injection of a step current pulse with the amplitude *I*_*step*_ = 0.147 can move the state from *s*_0_ to *s*_1_ and *s*_2_. (**b–d**) Time series of membrane potential (*v*) and external current (*i*_*e*_). Instantaneous threshold *θ*_*i*_ (red dotted lines) determines whether the injection of a brief current will cause state *s*_*i*_ to generate an action potential. The blue solid lines show the time series of state *s*_*i*_ without any stimulus, while the green dashed lines (red dash-dotted lines) demonstrate that the application of instantaneous current bring membrane potential of state *s*_*i*_ to below (above) threshold *θ*_*i*_. The durations of the step current injection to *s*_1_ and *s*_2_ are 7.429 and 12.5, respectively. The model is a classic FHN model with the following parameters: *τ*_*w*_ = 15, *a* = 1.25, *b* = 0.875.

The threshold voltage of an action potential is not clearly defined, and neuroscientists use varying criteria. The most commonly used threshold voltage determination is an *ad hoc* value of *dV*/*dt*^5^,^9^,^39^ obtained by visual inspection. All the criteria^1^,^2^,^3^,^39^ including this method are trying to determine a voltage value standing for the initial point of an AP in the already obtained AP time series, and produce some threshold variation^39^. To explain and predict the spike-threshold and its variation, we define the threshold voltage as *instantaneous threshold voltage*^18^, denoted as *θ*. The instantaneous threshold voltage is the voltage value that, when the membrane potential is instantaneously brought above or below it, will induce one AP or more (see Fig. 1). As opposed to the method in ref. 18, we do not limit the threshold to be a depolarized value, i.e., a hyperpolarized threshold due to inhibition is also acceptable. Theoretically, instantaneously shifting the membrane potential should be realized by injecting instantaneous currents (*i*_*e*_(*t*) = *qδ*(*t*)), and the instantaneous threshold voltage is searched by adjusting the strength of instantaneous current. In the application, a brief current injection is a close approximation of instantaneous current. From the dynamic system viewpoint, the instantaneous threshold voltages are defined by the separatrix, i.e., the boundary that separates two behavior modes (the membrane potential returning to the resting state after a small rise or a large excursion).

**Figure 2:**
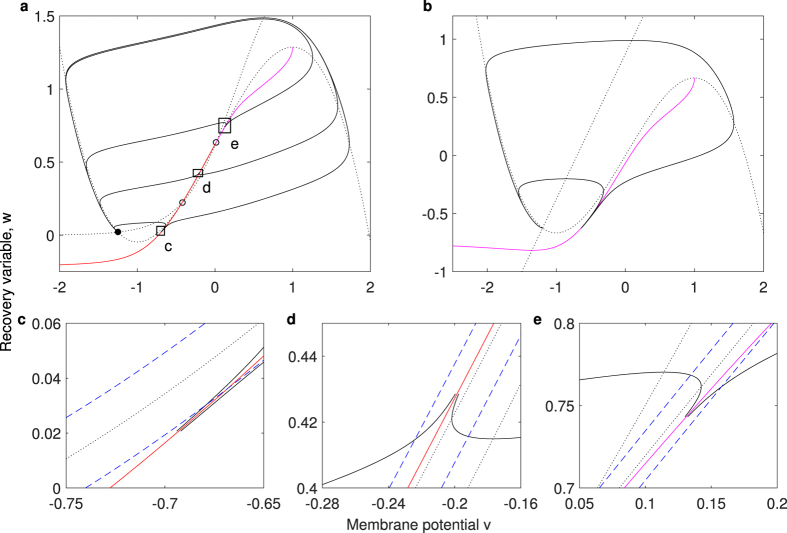
Separatrices determine threshold voltages in the state space of type I and type II neurons. Red and magenta solid lines are separatrices. The black solid lines are real trajectories, and the black dotted lines are nullclines (*dv*/*dt* = 0 and *dw*/*dt* = 0), respectively. (**a**) Separatrices of type I neuron: Boltzmann-FHN neuron model. The red separatrices represent the two stable manifolds of the saddle (left open circle), one extends to minus infinity while the other connects to the unstable node (right open circle). The magenta separatrix is in fact a trajectory that connects the unstable node and the fold point of cubic *v*-nullcline. Rectangles indicate the enlarged regions of (**c–e**). (**b**) Separatrix of type II neuron: classic FHN neuron model. There exists only one equilibrium that represents the resting state, and the canard-mechanism^29^ determines the threshold set (quasi-separatrix) of this type II neuron. (**c–e**) The enlarged regions of (**a**) show the coexistence of separatrices and the separatrices determine the thresholds rather than the *v*-nullcline or the curve in which *dv*/*dt* is equal to a certain value. Up/left and down/right blue dashed lines represent the curve that *dv*/*dt* equals −0.015 and 0.015, respectively.

As illustrated in Fig. 2, the different separatrix types for different neuron classes define the instantaneous threshold voltages well. As an excitable dynamic system, the neuron defined by equations (2) and (3) must possess at least one stable equilibrium. The stable equilibrium of a neuron is usually a resting state or sometimes a small oscillation^31^,^32^. From the viewpoint of a dynamical system, the resting state is a stable fixed point, and a small amplitude oscillation corresponds to a small limit cycle^31^,^32^. When more than one stable equilibrium exists, the attraction boundary of the resting equilibrium naturally forms the separatrix (see Fig. 2a,c,d). Usually, the stable manifolds of a saddle (the mathematical real-separatrix) play the threshold role and are called the STP by FitzHugh^26^. For those with only one stable equilibrium, the separatrix maybe the canard of a fold point^29^,^33^,^40^, which is called the quasi-separatrix^34^ (Fig. 2b). The threshold canard always exists and the threshold phenomenon is distinct when the time constants of the predominant fast and slow variables largely differ^29^,^33^. These phenomena were referred to as QTP by FitzHugh, and can be called *canard threshold phenomenon* (CTP) on the recent advances^29^,^33^,^40^. The Fenichel’s Theorem ensures the general existence of quasi-separatrix^29^,^30^,^33^,^41^. The typical type I and type II neurons belong to the type with more than one equilibrium and the type with only one equilibrium locally, respectively. However, CTP and STP can coexist in a type I neuron (Fig. 2a with enlarged view in c, d and e, e shows CTP). It should be noted that the *v*-nullcline or the curve that *dv*/*dt* equals a certain values is not the threshold voltage set, especially in Fig. 2d in which both initial values exhibit a negative *dv*/*dt*. The threshold voltage determined by an *ad hoc* large positive value of *dv*/*dt* is the indication that an AP has happened but is not a predictive criterion of whether an AP will be generated.

As described in the above paragraph, a separatrix is a set of special state points that determines the threshold for AP generation in the phase/state space. For a certain neuron, a separatrix is the function of the state variables and external current, but the specific form depends on parameters of channel gating variables *X*, such as maximum conductance 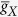 and reversal potential *E*_*X*_^16^. The full expression of the separatrix should be written as *S*(*v*, **X**; *i*_*e*_; **λ**) = 0, where **λ** denotes the vector of parameters. Focusing on the dynamic threshold of a specific neuron, we ignore the specific form of a separatrix and assume that a separatrix has a general form of *S*(*v*, **X**; *i*_*e*_) = 0, so the voltage values at the separatrix, *θ*(**X**; *i*_*e*_), are instantaneous threshold voltages. When the state of a neuron is changed by a stimulus from one side to the other of a separatrix in state space, the neuron switches between AP generation and subthreshold fluctuation. The crossing at different points of a dynamic separatrix caused by different stimuli bring about the spike-threshold variation measured. In addtiton, the separatrices of a neuron determine the possible variation scale of the spike threshold.

According to the general form of a separatrix, i.e., *θ*(**X**; *i*_*e*_), and the normal form of the time evolving equation of ion channel gating variables (equation(3)), we observe that the threshold varying versus time:


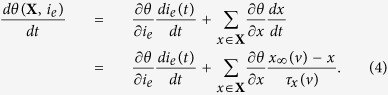


This equation indicates that the dynamic threshold depends on both the external current and the adaptation of all of ion channels, in principle. For neurons with DC injection or without external inputs, the first term in the right hand of the above equation is zero. Additionally, if one channel gating variable dominates the threshold dynamics, then equation (4) can be simplified as:


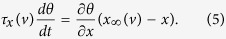


If ∂*θ*/∂*x* is a constant, i.e., thresholds are linearly dependent on the dominant gating variable (*θ*(*X*) ~ *kx*, here *k* is a constant), and the characteristic time (*τ*_*x*_(*v*)) is approximately a constant for sub-threshold currents, the above equation can be further transformed into the simplest threshold dynamic equation–equation(1). Equation (1) is directly assumed in refs 6, 9 and 15, while in ref. 16, it is deduced by a special condition that has no simple correspondence. Our derivation implies that equation (1) will fit well for the situations in which the threshold is linearly varied with the only one dominant modulating variable with an approximately constant characteristic time, in the process of DC injection or when no external current is presented.

Threshold states explained by separatrices are distinctly distinguished from and, at the same time, are associated with threshold parameters described by the bifurcation theory. The bifurcation theory describes different dynamic behavior caused by the variation of fixed parameters and does not take into account the initial states. The separatrix determines the threshold phenomenon of different initial values. Take the external current injection as an example; the bifurcation theory describes the threshold amplitude of DC and explains the rate coding of periodic repeated firing, while the separatrix framework determines the threshold voltages under subthreshold DC or transient inputs (e.g., step current) and relate to the temporal coding. However, parameters determine the specific form of a separatrix. The variation of parameters will quantitatively change the separatrix if no bifurcation occurs. Parameter thresholds determined by bifurcation change the form of the separatrix, and may even cause the separatrix to appear or disappear, e.g., saddle-node bifurcation in the type I neuron model^31^,^32^. When entering into the mode of repeated firing controlled by a global limited cycle, separatrices/state thresholds disappear. To completely predict the response of a neuron to the varying stimuli, we should combine both mechanisms.

Recent experiments and numerical simulations have attempted to elucidate the influence of the external stimulus on threshold voltages by defining threshold voltages as the post-stimulus threshold values^9^,^24^,^28^. The post-stimulus threshold is a unique case in that *i*_*e*_(*t* > *t*_*e*_) = 0 (*t*_*e*_ denote the time that the stimulus is off), i.e., the separatrix after the stimulus off is *θ*(**X**; 0). The separatrix is therefore the same as the old separatrix prior to the stimulus. The mechanism for post-stimulus threshold indicates that the changed state crosses the same separatrix. Separatrix-crossing in transient responses is different: the almost unaltered state was settled in the other side of the separatrix by moved separatrix.^29^,^34^ (*θ*(**X**; *I*_*e*_)). We emphasize the word ‘almost’, because the abrupt injected current does change the state; however, the magnitude of voltage shifting is far smaller than the shifting of *v*-nullcline (*I*Δ*t*/*C* ≪ *I*), when the switching duration Δ*t* is very short. In more general situations, the state and separatrix vary at the mean time with the continuous varying stimulus.

A voltage clamp fixes the membrane potential, but it changes other states of the membrane and therefore also alters the threshold. After a voltage clamp, AP generation is also determined by whether the clamped voltage exceeds the instantaneous threshold *θ*(**X**; 0). After any stimulus, if no AP is produced following the most recent deviation from the resting potential, then whether the system will produce an AP is determined by whether the state after the stimulus is turned off crosses the separatrix *θ*(**X**; 0).

In the following paragraphs, we apply the separatrix to different models and explain threshold phenomena using the separatrix-crossing mechanism. The separatrices, i.e., threshold sets, of these model are a point in the QIF model, a curve in 2D models, a plane or surface in 3D models and a hypersurface in the four dimensional HH model.

### Dynamic threshold point determined by stimulus

In the classic Integrate-and-Fire (IF) model, the threshold voltage is a fixed parameter. Recently, the dynamic threshold has been introduced using artificial mechanisms to predict the spiking series of real neurons^42^,^43^. Here, we demonstrate the dynamic threshold in a one-dimensional quadratic integrate-and-fire (QIF) model^31^. The separatrix in a QIF model is a point, which is limited by parameters but varies in response to varying stimulus strengths. The dimensionless equation of the QIF model is^31^


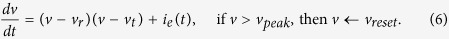


The parameters are related in that *v*_*peak*_ > *v*_*t*_ > *v*_*r*_ > *v*_*reset*_. When *i*_*e*_(*t*) = 0, the resting potential is *v*_*r*_ and the threshold is *v*_*t*_.

According to the bifurcation theory, the QIF model is topologically equal to the canonical type I neuron model and exhibits a saddle-node bifurcation if the DC current amplitude crosses the threshold value *I*_*rehobase*_^31^. *I*_*rehobase*_ is determined by the equation (*v*_*t*_ + *v*_*r*_)^2^ − 4(*v*_*r*_*v*_*t*_ + *I*_*e*_) = 0, i.e., the discriminant of the square equation *dv*/*dt* = 0. For a DC input with the amplitude *I*_*e*_, if *I*_*e*_ > *I*_*rehobase*_, the neuron periodically discharges; if *I*_*e*_ < *I*_*rehobase*_, the two real, unequal roots of equation *dv*/*dt* = 0 are two equilibria, threshold *θ* and resting potential *v*_*rest*_.

For the varying external current *i*_*e*_(*t*), replacing *I*_*e*_ with *i*_*e*_(*t*) leads to the dynamic threshold *θ*(*i*_*e*_) and dynamic resting potential *v*_*rest*_(*i*_*e*_) of:


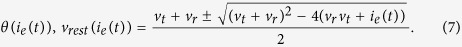


**Figure 3:**
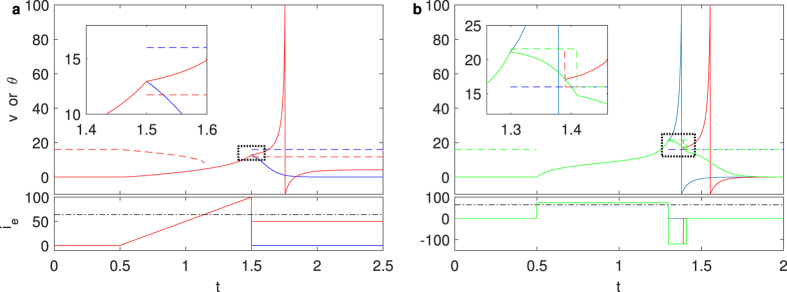
Dynamic threshold point varied with stimuli in the Quadratic Integrate-and-Fire model. (**a**) After a ramp current, an AP cannot be induced without stimulation (blue solid line); a subthreshold constant current that lower the threshold can elicit an AP (red solid line). The different behavior can be predicted by the threshold lines–corresponding dashed lines. Red lines represent the situation with subthreshold current *I*_*e*_ = 50. The black dash-dotted line represents the threshold current. (**b**) The rectangular pulse induced AP (blue solid line) can be prolonged (red solid line) or prevented (green solid line) by hyperpolarized current injection. Whether the membrane potential exceeds the corresponding threshold predicts whether the neuron will generate an action potential after the stimulus switching.

Both the threshold *θ*(*i*_*e*_) and resting potential *v*_*rest*_(*i*_*e*_) vary with the external current *i*_*e*_(*t*). The subthreshold depolarizing current elevates the resting potential and lowers the threshold, i.e., decrease the difference between the threshold and resting potential. The hyperpolarized current increases the difference between *θ*(*i*_*e*_) and *v*_*rest*_(*i*_*e*_), vice versa. When parameters are fixed in the QIF model, the threshold voltage is only related to the external stimulus. As displayed in Fig. 3a, the same membrane potential may be subthreshold with no stimulus or suprathreshold when a sufficiently strong current was injected. Once the membrane potential crosses the threshold, the neuron is in the process of AP generation. AP generation is a fast, but not instantaneous, process, and can be interrupted even during the depolarizing phase. After the moment the threshold is crossed, a strong and long enough hyperpolarized current injection, which elevates the threshold, may interrupt the firing process (see Fig. 3b). In a rigid sense, after the membrane potential exceeds the instantaneous threshold, the application of external current ensured a persistent increase in the difference between the membrane potential and the instantaneous thresholds determine the AP. To predict the generation of an AP, it is necessary to know both the subsequent stimulus and the present state of the neuron.

### Dynamic threshold curve in two-dimensional models

As a one-dimensional system, the QIF model has a threshold value that varies with an external stimulus. In the similar 2-dimensional Izhikevich model, the threshold expands to a curve^31^. The threshold curves in a 2-dimensional model have been demonstrated in many dynamic systems, such as the FitzHugh-Nagumo (FHN) model^19^,^31^,^44^, the reduced 2-dimensional HH model^44^, the Morris-Lecar (ML) model^19^,^34^, etc.^19^ However, the quantitative form of the separatrix cannot be precisely determined in these models. We present the analytic separatrix in a bi-dimensional model with piecewise linear nullclines and explain, in detail, the threshold phenomena that are determined by the separatrix-crossing mechanism for voltage clamping and other stimuli. We also deduce the first-order differential equation that describes the dynamic threshold variation versus time. This 2D PWL model is analytically solvable and can be regarded as a modified FHN model.

**Figure 4:**
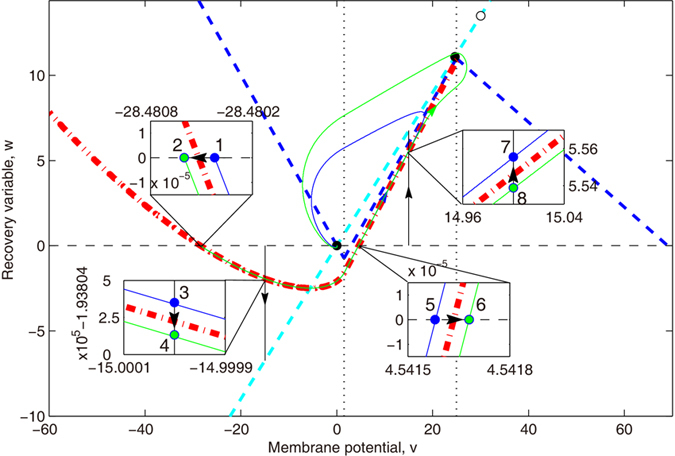
Separatrices and the separatrix-crossing mechanism after voltage clamp in the state plane of a 2D PWL model. Blue and cyan dashed straight-lines represent the *v*-nullcline and *w*-nullcline, respectively. Black dots and circles are three fixed points; the left one (stable spiral, black dot) is real, while the middle one (stable spiral, black dot) and the right one (saddle, black circle) are virtual. Wide red dash-dotted curves delineate the global separatrices, the right straight one in the middle region is a real separatrix (stable manifold) of the saddle, while the left winding one is a trajectory connected to the real separatrix. Blue and green solid lines are real trajectories. Black lines show the voltage clamp process, the dashed one demonstrates the ideal instantaneous voltage shift and the solid two with an arrow marking the time evolution show the voltage holding after the sudden shift. Initialized at state points 1, 3, 5 or 7, the neuron will follow the blue trajectory and has a small peak, which is considered a subthreshold fluctuation; initialized at the state points 2, 4, 6 or 8 (on the other side of separatrix–firing region), the peak of the membrane potential will be large and will be considered an AP. The difference between 1 and 2 (5 and 6) is whether the sudden shift of membrane potential from resting potential cross the separatrix, while the difference between 3 and 4 (7 and 8) is whether the continuous voltage holding crosses the separatrix.

As shown in Fig. 4, the separatrices determine the thresholds of our 2D PWL model. The real separatrix is a straight line that is precisely determined by the intersecting point (saddle) of the middle segment of the *v*-nullcline and *w*-nullcline. The separatrix in the hyperpolarized voltage region is in fact the trajectory connected to the real separatrix.

The real separatrix of our 2D PWL model is a straight line in the *v* − *w* state plane


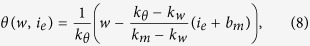


where *k*_*w*_, *k*_*m*_ and *b*_*m*_ are parameters; and *k*_*θ*_ is the slope rate of the separatrix which depends on the parameters including the time constant *τ*_*w*_ (see Section Methods). The threshold is linearly related to the recovery variable *w*. Because *k*_*θ*_ > *k*_*m*_ > *k*_*w*_ (see Section Methods), we can infer that the threshold decreases as the strength of the depolarizing current increases. The *i*_*e*_ increment linearly moves the separatrix (a straight line) up in the *v* − *w* plane. Therefore, an external current linearly changes the thresholds.

In the following paragraphs of this subsection, we will demonstrate that separatrix-crossing determines threshold parameters and threshold voltage variation after voltage clamping, step current and ramp current injections.

We demonstrate the separatrix-crossing mechanism of threshold variation after voltage clamp (*θ*(*w*, 0)) in the state plane of our 2D PWL model in Fig. 4. For a clear demonstration, we only use two different trajectories that originate from the state points 1 and 2 in the phase plane with the former (blue solid line) on the non-firing zone and the latter (green solid line) on the other side of the separatrix (red broad dash-dotted line) representing the firing zone. From Fig. 4, we observe the threshold phenomenon in rapid voltage shift from resting potential: the instantaneous voltage shift to state 2 or 6 horizontally across the separatrix from the resting zone to the firing region and thus an AP was generated. Conversely, there is no firing for the shift to state 1 or 5. The continuous voltage holding vertically evolves and then crosses the separatrix in the state plane, thereby determines another threshold phenomenon: the threshold timing of a voltage clamp at a fixed voltage. For example, if a depolarized voltage is held at 15 *mV* and the holding time is lengthened, the neuron will pass through state point 7 to state point 8, which prevent AP generation. Inversely, if a hyperpolarized voltage is held at −15 *mV*, an increased clamping time will cause an AP generation (state 4) while a shorter clamping will not (state 3). The long voltage clamping at hyperpolarized potential facilitates AP generation. Therefore, the winding separatrix around the resting state in the hyperpolarized region results in post-inhibitory facilitation.

**Figure 5:**
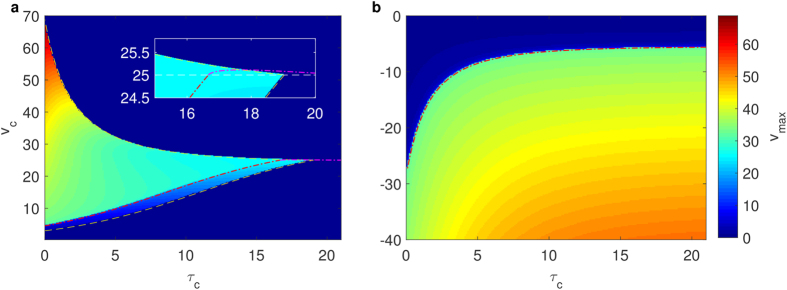
Maximum voltages indicate threshold clamping voltages and times in the 2D PWL model. Numerical results (colored patches) fit the analytical results (colored lines). (**a**) Maximum voltages after depolarization voltage clamp. For a fixed clamping voltage *v*_*c*_, threshold clamping duration (*τ*_*c*_) in the low voltage region (analytical results are indicated by the magenta dash-dotted line) indicate that a long depolarized voltage suppresses AP firing, while threshold clamping duration for a high voltage well fit the curve *dv*/*dt* = 0 (yellow lines) which indicates that longer clamping potential time-series is the lack of a depolarizing phase of AP. (**b**) Maximum voltages of hyperpolarized voltage clamping. For a constant voltage, inverse with the depolarized situation, a short voltage clamping cannot induce an AP but a longer voltage clamping can. The threshold voltage for a fixed clamping time is a monotonic increasing function of clamping duration.

As shown in Fig. 5, the threshold voltages and timing that are determined by separatrix-crossing are clearly exhibited by the maximum rising voltages (*V*_*max*_) after voltage clamps with clamped voltage *v*_*c*_ and clamping duration *τ*_*c*_. Comparing two subfigures in Fig. 5, we find that a long voltage holding at a depolarized potential hampers AP generation. Conversely, a long voltage holding at a hyperpolarized potential facilitates AP generation. This phenomenon is caused by separatrix-crossing from the firing zone to the subthreshold zone during depolarized voltage holding and *vice versa* during hyperpolarized voltage holding, as demonstrated in Fig. 4 (from state 8 to state 7 and from state 4 to state 3, respectively). The border that is indicated by the color in the low voltage region (*v* < 25 *mV*) in Fig. 5a represents the normally mentioned threshold voltage and correctly fits the curve *v*_*c*_ = *θ*(*v*_*c*_, *τ*_*c*_), which is the projection of the real-separatrix in the *τ*_*c*_ − *v*_*c*_ phase plane. The border in the high voltage region corresponds to the line *dv*/*dt* = 0. Additionally, in Fig. 5b, the separatrix projection in the hyperpolarized voltage region (left region in phase space) precisely limits the domain of the firing region of the numerical results.

Similar to voltage clamping, current injection, such as rectangular and ramp current pulses, will push the state across the separatrix if the pulse duration is long enough (see Supplementary Fig. S1). The threshold pulse strengths and durations, which are the projection of separatrices in the *v* − *w* phase plane, will form a threshold boundary in the parameter plane.

According to eqs (4) and (8), we can deduce the first order threshold equation


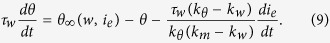


If *di*_*e*_/*dt* = 0, i.e., *i*_*e*_(*t*) ≡ *I*_*e*_, our 2D PWL model satisfies the conditions of equation (5), and we obtain the same threshold equation reported in refs 9 and 18.

Our PWL model is more like a type III neuron according to Hodgkin’s classification, as it can not generate periodic APs. However, if we set the slope of *w*-nullcline to be larger than the middle segment of *v*-nullcline, the PWL model is capable of firing periodically and of having a type II *f*-*I* curve. Additionally, the separatrix is an unstable manifold of the unstable node of nullclines in the middle region (See Fig. S2.). Our 2D PWL models have only one real equilibrium and lack fold points (*v*-nullcline where has *dw*/*dv* = 0), and more important is the distinctiveness of threshold phenomena depends on the time constant (see Fig. S3.). The threshold phenomena in these models essentially belong to CTP (QTP). However, it is interesting that the *v*-nullcline is discontinuous (DTP), and the threshold is determined by a real-separatrix (STP) which locates in the layer of canards (CTP).

### Threshold surface in three-dimensional neuron models

Here, we extend the two-dimensional PWL model to a three-dimensional model, which provides an example of three-dimensional phase space analysis of threshold phenomena.

**Figure 6:**
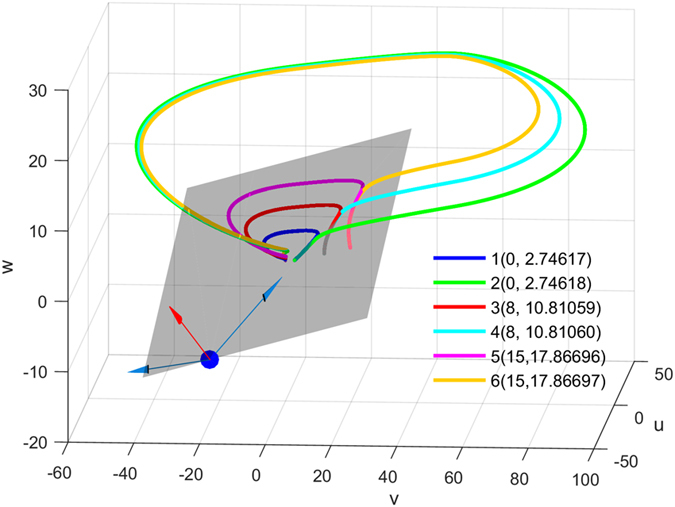
Separatrix determines the threshold voltages in a three-dimensional PWL model. Trajectories 1, 3 and 5 (blue, red and magenta lines) behind the threshold plane and trajectories 2, 4 and 6 (green, cyan and yellow lines) before the threshold plane exhibit small difference in initial values; however, they have great behavior difference, subthreshold fluctuation or firing. The gray transparent plane, which divide the space into subthreshold and suprathreshold regions, is a separatrix determined by stable (red arrow) and unstable (blue arrow) eigenvectors of an equilibrium (blue point). The legend shows the initial coordinates in the form (*v*_0_, *u*_0_) (*w*_0_ = 0 is ignored).

As shown in Fig. 6, a plane of threshold states that separates the 3-dimensional space into two parts defines the firing or non-firing region in the middle region. The separatrix in this 3D PWL model is a real separatrix that is actually a plane determined by the stable and unstable eigenvectors. For dynamic analysis, see Supplementary Note S1 and Supplementary Fig. S4.

The separatrix, i.e., a threshold plane in the middle region is


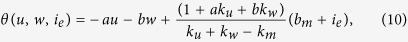


where *a*, *b*, *k*_*u*_, *k*_*w*_ and *k*_*m*_ are parameters (see Methods section). The threshold variation equation versus time can be obtained as


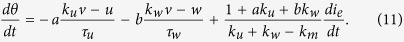


The two characteristic time constants, *τ*_*u*_ and *τ*_*w*_, prevent the simplification of equation (11) into equation (1) if the difference of *τ*_*u*_ and *τ*_*w*_ is neither very large nor very small.

The threshold variation phenomena of post-stimulus, such as a voltage clamp, step current or ramp current, are also determined by separatrix-crossing in the 3D PWL model. Additionally, it is a natural extrapolation that similar higher dimensional PWL models may have separatrices in the form of hyperplanes.

Similar to our 2D PWL model, the threshold in this 3D PWL model is essentially belong to CTP, but the threshold set can be expressed as the real-separatrix which is in the layer of canards. As shown in Figure S5, we also provide the continuous threshold hypersurface in 3D FHN model, which is determined by a quasi-separatrix in 3D state space. The fold point in Fig. 2 expands to a fold curve (see Fig. S5, the solid black straight line). The separatrix comprises threshold manifolds passing through the fold points in the fold curve.

### Threshold hypersurface in the HH model

**Figure 7:**
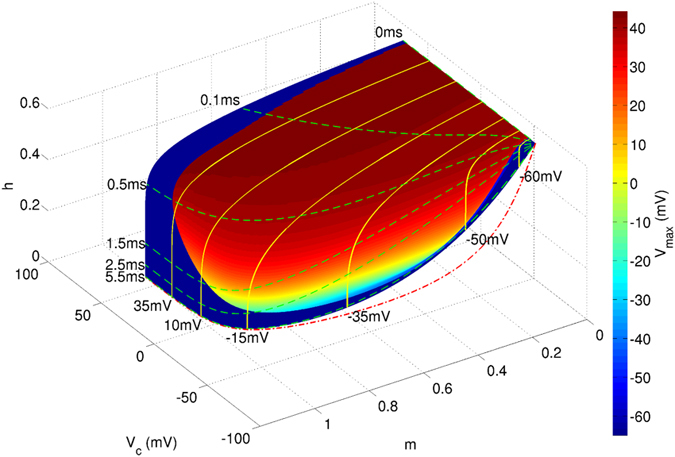
The separatrix-crossing mechanism of the HH model after voltage clamp in the *V*–*m*–*h* state space. Colors on the voltage clamping surface indicate maximum voltages after voltage clamping. The clear boundary between blue and other colors is the numerical approximation of separatrix on a voltage clamping surface in the *V*-*m*-*h* phase space. The red dash-dotted line reveals the asymptote when the voltage clamping time is infinity. Yellow solid lines show voltage clamp processes at several voltages (*V*_*c*_-lines) and the green dashed lines indicate the same duration after voltage clamp (*τ*_*c*_-lines); all are marked with a number, showing corresponding values at the end. The separatrix in *V*-*m*-*h* space is part of the global separatrix in four dimensional phase space and divides the voltage clamping surface into firing and no firing regions. A *V*_*c*_-line shows that the state of the neuron continuously changes, crosses the separatrix and comes into the no firing region as the clamping time *τ*_*c*_ increases.

We numerically demonstrate the separatrix-crossing mechanism in the classic HH model after voltage clamps, as shown in Fig. 7. The maximum depolarizing voltage *V*_*max*_ (indicated by color) after a voltage clamp under the corresponding *V*_*c*_ (clamped voltage) and *τ*_*c*_ (clamping duration) is mapped onto the voltage clamping surface. The clear boundary of the maximum voltage is a numerical approximation of the separatrix, which shows distinct characteristics in subthreshold and firing regions. The similar separatrices can be numerically plotted in other 3D state spaces (such as the *m*-*h*-*n* space, see Supplementary Fig. S6). The separatrix line in the voltage-clamp surface is part of a high-dimensional separatrix that intersects the voltage clamping surface.

Voltage clamp transient processes at several different potentials are displayed as yellow solid curves with the potential marked at the end (*V*_*c*_-lines) in Fig. 7, while the green dashed lines with time marks (*τ*_*c*_-lines) show the voltage clamping duration. The red dash-dotted line indicates the asymptotic value as time approaches infinity under different clamp voltages (*m*_∞_(*V*) and *h*_∞_(*V*)). As *V*_*c*_-lines indicate, when the voltage is clamped at a certain value, an immediate release causes the system to remain in the firing zone, allowing for the generation of an AP. Conversely, if being held at a voltage for a long time, the system will cross the separatrix into the subthreshold zone, and APs cannot be generated.

**Figure 8:**
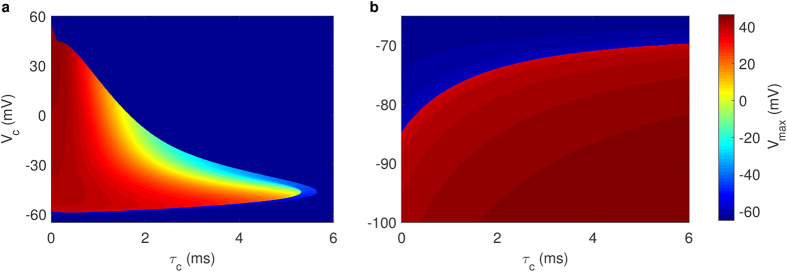
Maximum voltages indicate threshold clamping voltages and times in the classic HH neuron. (**a**) Maximum voltages after depolarized voltage clamp. For a fixed clamping voltage *V*_*c*_, as the clamping duration *τ*_*c*_ increases, the peak of the action potential decreases. Extended voltage holding inhibits AP generation after voltage clamping. The border in the low voltage region displays the normal threshold and the border in the high voltage region is determined by *dV*/*dt* = 0 (see Supplementary Fig. S8). (**b**) Maximum voltages of hyperpolarized voltage clamps. For a constant voltage, inverse with the depolarized situation; the short time clamps cannot induce an AP while the long-enough ones can. As opposed to the depolarized situation, the threshold voltage for a fixed clamping time is a monotonic increasing function.

As seen in the PWL models, whether the HH neuron will generate an action potential after voltage clamping is determined by whether the clamped voltage (*V*_*c*_) exceed the instantaneous threshold, *θ*. We numerically show the scenarios of threshold variation during several voltage clampings with varying clamped voltages and clamping durations in Fig. S7. The intersecting points of *V*_*c*_ and *θ* form the clear threshold border in the low voltage region of Fig. 8, i.e., *V*_*c*_ = *θ*(*V*_*c*_, *τ*_*c*_; 0) as *m*, *h* and *n* is the function of *V*_*c*_ and *τ*_*c*_ according to equation (29)). The similarity of maximum voltages versus clamping voltages and durations in the 2D PWL model and the HH model indicate similar separatrix-crossing mechanism, but the differences in the details indicate the complex interaction of different gating of ion channels in the HH model (Supplementary Fig. S8).

According to the general equations (4) and (29), the instantaneous threshold voltage variation in the HH model following a voltage clamp (*θ*(*V*_*c*_, *τ*_*c*_; 0)) is determined by


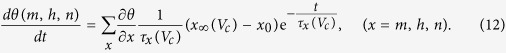


From the above equation, we observe that in principle, all channels and their subunits exert effects on the threshold variations because they are all coupled to the membrane potential; however, the significance is determined by weights of both the time constant *τ*_*x*_(*V*_*c*_) and ∂*θ*/∂*x*. Additionally, if the external stimulus is not a voltage clamp, e.g., ramp current, the ‘time constant’ *τ*_*x*_(*V*) for each gating variable also varies with the membrane potential, and therefore, the temporal evolution of post-stimulus threshold is more complex.

## Discussion

Highly variable spiking thresholds are observed both *in vitro*^6^,^9^,^10^ and *in vivo*^2^,^3^,^4^,^5^,^6^,^7^. Recent studies concerning the threshold variation of neural spikes demonstrate three distinct characteristics. First, as the threshold is stimulus dependent, most researchers use the threshold after the stimulus is off^9^,^24^,^28^. In the current study, we show that the separatrix-crossing mechanism is universal for post-stimulus^9^,^24^,^28^ (*θ*(**X**; 0)) and transient^29^,^34^ (*θ*(**X**; *I*_*e*_)) threshold phenomena. Second, because the threshold is not a single value, several studies have extended the threshold point to a threshold curve^19^,^28^. In fact, the separatrix concept has been proposed since the 1950s^26^,^44^, and similar hypotheses for general threshold also have recently been reintroduced^19^,^27^,^28^. In the current paper, we demonstrate that different types of separatrices generally exist in excitable neuron models and systematically show that separatrix-crossing mechanism determines threshold phenomena in example models, especially the 1-, 2- and 3-dimensional models with analytic separatrices. We also found that the singular-point threshold and canard threshold phenomena coexist in type I neuron which have previously been considered to only have singular-point threshold phenomena. Third, researchers attempt to simplify the threshold problem and construct quantitative threshold equations^9^,^16^,^17^,^19^. Incorporating dynamic thresholds into the IF model was shown to be a very efficient way to predict spike events in real neurons^38^. Researchers also have simplified the threshold variation to a threshold equation^16^,^17^,^18^,^19^. Here, we deduced a detailed threshold evolving equation versus time, which can be simplified to the previously assumed equation^6^,^9^,^15^,^16^,^17^,^18^,^19^ for the simplest case.

Separatrix-crossing framework includes different ion channel gating variables with different characteristic times. The ion channel subunits with long characteristic times may induce the threshold variation related to previous spikes^23^,^45^. According to ref. 33, the synaptic current with a long decay can determine the cessation of rebound spiking, which can not be explained by inhibitory current, as if the synaptic current was added using a differential equation of an independent variable. Thus, we infer that the separatrix in full state space should include the threshold modulation mechanisms over a long time, if all of the factors influencing the threshold are included in the differential equations. If the difference in characteristic time scales between dominant slow and fast variables is not large, intermediate-amplitude AP will be generated and threshold phenomena maybe ambiguous^31^ (QTP), which is the the reason why the quasi-separatrix is always mentioned as ‘not well-defined threshold’ in neuron models^31^,^32^,^38^. However, considering that the type II neurons are functional in nervous systems, so it is reasonable to infer that either these neurons do have large enough differences of characteristic time scales, or other factors such as noise are able to overcome this weakness. In addition, the threshold voltages can still be defined by the quasi-separatrix even though the threshold phenomena are not distinct.

In the present framework of separatrix-crossing, the noise or highly fluctuating inputs are not included. In our framework, the noise and the fluctuating inputs do not only change the state of the neuron but also vary the threshold. However, because that the threshold is instantaneous, it is possible to extend the framework to including the noise or fluctuating inputs, which cause instantaneous random variation of the threshold and state. Khovanov *et al*.^35^ and Franović *et al*.^36^,^37^ have studied how the external and internal noise cause a spontaneous spike by considering quasi-separatrix (‘ghost-separatrix’^35^) or separatrix-like boundary (‘terminating boundary’^36^,^37^) using different methods in different neuron models, and a further exploration is worthwhile.

Although the full explanation for spike-thresholds (especially the threshold of spontaneous spikes) should resort to the model with noise, the present framework can still provide some qualitative understanding. It has been found that the threshold voltage is positively correlated with the membrane potential (*V*)^1^,^2^,^3^ and negatively correlated with the rising rate of membrane potential (*dV*/*dt*) before the spike^2^,^3^,^45^. In state space of deterministic models, long enough dwelling at a membrane potential without stimulation before the spike (250 ms in refs 1, 2, 3) implies that the membrane state is close to the asymptotic state of long voltage holding (the *w*-nullcline). The corresponding instantaneous threshold does increase with the membrane potential in the *w*-nullcline (see Fig. S9). The negative correlation between threshold and the rising rate of membrane potential is obvious^24^,^25^. As displayed in both Figs S1 and S9, the stronger currents cause trajectories on which the gating variables varied less for the same *v* increment than that of the weaker currents. The weaker adaptation of gating variables, especially the inactivation of *Na*^+^ channel and activation of *K*^+^ channel, induced by a more synchronous stimulus brings a lower threshold.

The threshold variability and the rapidity of onset of AP have inspired a recent debate^4^,^5^,^10^,^46^,^47^,^48^,^49^ concerning whether the two characteristics can be satisfied at the same time by using the classic HH model^10^,^47^. Two explanations that try to reconcile the two conflicting facts concern more than one compartment^47^,^50^. In a multi-compartment model, the thresholds of each compartment are also affected by the neighbor’s states, such as the lateral current^47^ and the relative size of the soma and axon^50^. It has been demonstrated that the morphology and distribution of ion channels can affect the thresholds of a neuron, especially the axon initial segment, which has a very high sodium channel density^10^,^47^,^51^. How will the separatrices of compartments determine the threshold of the neuron? Our simple PWL models with precise thresholds provide applicable models that are suitable for future exploration of these concepts.

In summary, by introducing the general separatrix concept, we demonstrate that the separatrix generally exists in excitable neurons, and determine threshold voltages, threshold voltage variation and post-stimulus threshold parameters for AP generation. We brought forward the idea that threshold phenomena can be classified into two general types with different mechanisms, the *parameter threshold*, described by the bifurcation theory, and the *state threshold*, explained by separatrix-crossing. The separatrix-crossing mechanism also determines the threshold of timing-related parameters of independent variables. We also naturally deduced the threshold variation equation versus time and transformed it into the simple form used by previous researchers^6^,^9^,^15^,^16^,^17^,^18^ for a simpler case. Separatrix and separatrix-crossing-determined threshold phenomena were demonstrated and verified in example models. The separatrix-crossing mechanism and models we proposed will benefit further investigation of threshold variability on other factors, such as noise, morphology, passive membrane properties and the spatial distribution of the ion channels of neurons.

## Methods

### Classic FHN model and Boltzmann-FHN model

The classic FHN model is described by differential equations:


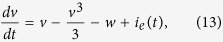



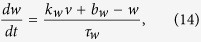


where *v* represents membrane potential, *w* is a recovery variable and *i*_*e*_(*t*) is time-varying injected external current. To demonstrate of canard threshold phenomenon well, we let *τ*_*w*_ = 15, *k*_*w*_ = 1.25, *b*_*w*_ = 0.875 and *i*_*e*_(*t*) ≡ 0. The neuron belongs to type II neuron.

Similar to ML-FHN neuron model used in ref. 29, we modified equation (14) of recovery variable *w* to:


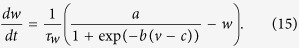


As the nullcline of *w* is a Boltzmann function, we call it as ‘Boltzmann-FHN model’. To show three separatrices determined by saddle and canard phenomenon well, we take *τ*_*w*_ = 8, *a* = 2, *b* = 3, *c* = 0.27 and *i*_*e*_(*t*) ≡ 0.62. The model is a type I neuron.

To show how canard trajectories constitute a quasi-separatrix in three dimensional state space (Fig. S5), we also extend the classic FHN model to be three dimensional by adding a slow recovery variable *u* similar as *w*: *u* is substracted in equation (13) and *du*/*dt* follows the same form of equation (14). The parameters for 3D FHN model in Fig. S5 are as follow: *τ*_*w*_ = 25, *k*_*w*_ = 1.25 and *b*_*w*_ = 0; *τ*_*u*_ = 15, *k*_*u*_ = 1.25 and *b*_*u*_ = 0; *i*_*e*_(*t*) ≡ −2.5.

### Two-dimensional piecewise linear model

The piecewise linear model is named for its piecewise linear nullclines in the phase space. The general form of the model we used in this paper is:


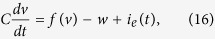



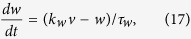


where *f*(*v*) is a piecewise linear function of *v* (membrane potential), *w* represents the recovery current, *C* is the membrane capacitance, *τ*_*w*_ = 5 is the time constant of *w*, and *k*_*w*_ = 0.45 is a coefficient. As a model demonstrate threshold phenomena in principle, the equations is dimensionless, so do the equations of 3D PWL model.

We take *f*(*v*) as a piecewise linear function:


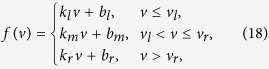


Slope rates of left, middle and right segments are represented as *k*_*l*_ = −0.5, *k*_*m*_ = 0.5, and *k*_*r*_ = −0.25, respectively, whereas the intercepts are *b*_*l*_ = 0, *b*_*m*_ = −1.5 and *b*_*r*_ = 17.25, and the parameters that separate the definition scales are *v*_*l*_ = 1.5 and *v*_*r*_ = 25.0.

According the derivation in Supplementary Note S1, the threshold voltages is:


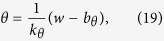


where *k*_*θ*_ is the slope rate of the threshold and is equal to 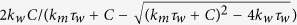, and *b*_*θ*_ = (*i*_*e*_ + *b*_*m*_)(*k*_*w*_ − *k*_*θ*_)/(*k*_*w*_ − *k*_*m*_) is the intercept. The threshold boundary by projecting the separatrix into the plane of clamped voltage versus clamping time (*τ*_*c*_ − *v*_*c*_) is as follow:


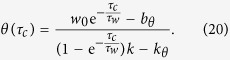


### Three-dimensional piecewise linear model

We extended the two-dimensional piecewise linear model to three dimensional to show the separatrix in the three dimensional phase space:


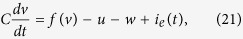



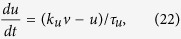



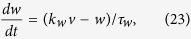


where *f*(*v*) is a piecewise linear function of membrane potential *v*, *f*(*v*) has the same form as in formula (18), *u* and *w* are the recovery variables, *τ*_*u*_ (*τ*_*w*_) is the time constant of *u* (*w*), and *k*_*u*_ > 0 (*k*_*w*_ > 0) is a coefficient.

Parameters of *f*(*v*) are the same as in the 2D PWL model, with the exception of *k*_*m*_ = 0.95, *b*_*m*_ = −2.175, *b*_*r*_ = 57.825, and *s*_*r*_ = 50.0. The slope rates are *k*_*u*_ = 0.45 and *k*_*w*_ = 0.6. Time constants are *τ*_*u*_ = 5.0 and *τ*_*w*_ = 10.0.

See the derivation in Supplementary Note S2, the explicit equation of the threshold can be written as:


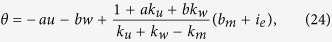


where 
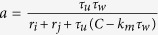
 and 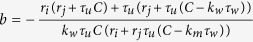 with *r*_*i*_ and *r*_*j*_ being the roots of Supplementary equation (S3). Although two threshold planes exist, only the plane shown in Supplementary Fig. S4 is located properly to serve as the threshold for this 3-dimensional PWL neuron.

### Classic Hodgkin-Huxley model

The equations of the classic HH model are^21^


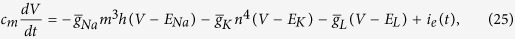



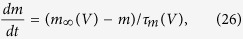



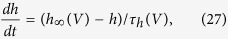



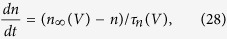


where *τ*_*x*_(*V*) = 1/(*α*_*x*_(*V*) + *β*_*x*_(*V*)) (*x* represents *m*, *h* or *n* here and in the following equations and paragraphs) is the time constant of *x*, and *x*_∞_(*V*) = *α*_*x*_(*V*)/(*α*_*x*_(*V*) + *β*_*x*_(*V*)) is the asymptotic value of *x* when time approaches +∞. The rate functions *α*_*x*_ and *β*_*x*_ are:


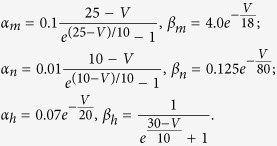


Reversal potentials are *E*_*Na*_ = 50 *mV*, *E*_*K*_ = −77 *mV* and *E*_*L*_ = −54.4 *mV*.

The neuronal membrane potential is clamped to the set voltage by an ideal voltage-clamp protocol. Briefly, the voltage is set to be the clamped voltage while the other equations are allowed to evolve. By setting *V* to be constant in equations (26)–(28), 32, 33, we obtain:


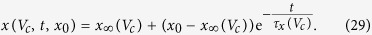


This equation describes the asymptotic transient process of gating variable *x* when the neuron is clamped to the voltage *V*_*c*_. Thus, we analytically calculate channel states *x*(*V*_*c*_,*τ*_*c*_) for the initial states *x*_0_. *x*(*V*_*c*_,*τ*_*c*_) is the new initial values to determine whether the neuron will firing or not following voltage clamping. Both the resting state and the subsequent evolution after voltage clamping were numerically calculated using the 4th-order Runge-Kutta method. The resting state was saved after 200 *ms* of running the model without any stimulation. After the voltage clamp, we did not provide other stimulus, thereby making the HH model an autonomous system again.

## Additional Information

**How to cite this article**: Wang, L. *et al*. Spike-Threshold Variability Originated from Separatrix-Crossing in Neuronal Dynamics. *Sci. Rep*. **6**, 31719; doi: 10.1038/srep31719 (2016).

## Supplementary Material

Supplementary Information

## References

[b1] Azouz R. & Gray C. M. Cellular mechanisms contributing to response variability of cortical neurons *in vivo*. J. Neurosci. 19, 2209–2223 (1999). 10066274 10.1523/JNEUROSCI.19-06-02209.1999 PMC6782570

[b2] Azouz R. & Gray C. M. Dynamic spike threshold reveals a mechanism for synaptic coincidence detection in cortical neurons *in vivo*. PNAS 97, 8110–8115 (2000). 10859358 10.1073/pnas.130200797 PMC16678

[b3] Azouz R. & Gray C. M. Adaptive coincidence detection and dynamic gain control in visual cortical neurons *in vivo*. Neuron 37, 513–523 (2003). 12575957 10.1016/s0896-6273(02)01186-8

[b4] Naundorf B., Wolf F. & Volgushev M. Unique features of action potential initiation in cortical neurons. Nature 440, 1060–1063 (2006). 16625198 10.1038/nature04610

[b5] Muñoz F. & Fuentealba P. Dynamics of action potential initiation in the GABAergic thalamic reticular nucleus *in vivo*. PLoS ONE 7, e30154 (2012). 22279567 10.1371/journal.pone.0030154 PMC3261188

[b6] Farries M. A., Kita H. & Wilson C. J. Dynamic spike threshold and zero membrane slope conductance shape the response of subthalamic neurons to cortical input. J. Neurosci. 30, 13180–13191 (2010). 20881137 10.1523/JNEUROSCI.1909-10.2010 PMC2966473

[b7] Howard M. A. & Rubel E. W. Dynamic spike thresholds during synaptic integration preserve and enhance temporal response properties in the avian cochlear nucleus. J. Neurosci. 30, 12063–12074 (2010). 20826669 10.1523/JNEUROSCI.1840-10.2010 PMC3390778

[b8] Wilent W. B. & Contreras D. Stimulus-dependent changes in spike threshold enhance feature selectivity in rat barrel cortex neurons. J. Neurosci. 25, 2983–2991 (2005). 15772358 10.1523/JNEUROSCI.4906-04.2005 PMC6725135

[b9] Higgs M. H. & Spain W. J. Kv1 channels control spike threshold dynamics and spike timing in cortical pyramidal neurones. J. Physiol. (Lond.) 589, 5125–5142 (2011). 21911608 10.1113/jphysiol.2011.216721 PMC3225669

[b10] McCormick D. A., Shu Y. & Yu Y. Neurophysiology: Hodgkin and huxley model still standing? Nature 445, E1–E2; discussion E2–E3 (2007). 17203021 10.1038/nature05523

[b11] Wallach A. & Marom S. Interactions between network synchrony and the dynamics of neuronal threshold. J. Neurophysiol. 107, 2926–2936 (2012). 22402648 10.1152/jn.00876.2011

[b12] Kuba H., Ishii T. M. & Ohmori H. Axonal site of spike initiation enhances auditory coincidence detection. Nature 444, 1069–1072 (2006). 17136099 10.1038/nature05347

[b13] Kuba H. & Ohmori H. Roles of axonal sodium channels in precise auditory time coding at nucleus magnocellularis of the chick. J. Physiol. 587, 87–100 (2009). 19001045 10.1113/jphysiol.2008.162651 PMC2670025

[b14] Yi G.-S., Wang J., Tsang K.-M., Wei X.-L. & Deng B. Input-output relation and energy efficiency in the neuron with different spike threshold dynamics. Front. Comput. Neurosci. 9 (2015). 10.3389/fncom.2015.00062 PMC4444831 26074810

[b15] Hill A. V. Excitation and accommodation in nerve. Proc. R. Soc. Lond., Ser. B, Biol. Sci. 119, 305–355 (1936).

[b16] Platkiewicz J. & Brette R. A threshold equation for action potential initiation. PLoS Comput. Biol. 6, e1000850 (2010). 20628619 10.1371/journal.pcbi.1000850 PMC2900290

[b17] Platkiewicz J. & Brette R. Impact of fast sodium channel inactivation on spike threshold dynamics and synaptic integration. PLoS Comput. Biol. 7, e1001129 (2011). 21573200 10.1371/journal.pcbi.1001129 PMC3088652

[b18] Fontaine B., Peña J. L. & Brette R. Spike-threshold adaptation predicted by membrane potential dynamics *in vivo*. PLoS Comput Biol. 10, e1003560 (2014). 24722397 10.1371/journal.pcbi.1003560 PMC3983065

[b19] Tonnelier A. Threshold curve for the excitability of bidimensional spiking neurons. Phys. Rev. E 90, 022701 (2014). 10.1103/PhysRevE.90.022701 25215752

[b20] Hodgkin A. L., Huxley A. F. & Katz B. Measurement of current-voltage relations in the membrane of the giant axon of loligo. J. Physiol. 116, 424–448 (1952). 14946712 10.1113/jphysiol.1952.sp004716 PMC1392219

[b21] Hodgkin A. L. & Huxley A. F. A quantitative description of membrane current and its application to conduction and excitation in nerve. J. Physiol. 117, 500–544 (1952). 12991237 10.1113/jphysiol.1952.sp004764 PMC1392413

[b22] Gutierrez C., Cox C. L., Rinzel J. & Sherman S. M. Dynamics of low-threshold spike activation in relay neurons of the cat lateral geniculate nucleus. J. Neurosci. 21, 1022–1032 (2001). 11157087 10.1523/JNEUROSCI.21-03-01022.2001 PMC6762305

[b23] Harrison P. M., Wall M. J. & Richardson M. J. Slow sodium-channel inactivation underlies spike threshold variability. BMC Neurosci. 14 (Suppl 1), P322 (2013).

[b24] Wester J. & Contreras D. Biophysical mechanism of spike threshold dependence on the rate of rise of the membrane potential by sodium channel inactivation or subthreshold axonal potassium current. J. Comput. Neurosci. 35, 1–17 (2013). 23344915 10.1007/s10827-012-0436-2 PMC3683126

[b25] Yi G.-S., Wang J., Tsang K.-M., Wei X.-L. & Deng B. Biophysical insights into how spike threshold depends on the rate of membrane potential depolarization in type I and type II neurons. PLoS ONE 10, e0130250 (2015). 26083350 10.1371/journal.pone.0130250 PMC4471164

[b26] FitzHugh R. Mathematical models of threshold phenomena in the nerve membrane. Bull. Math. Biophys. 17, 257–278 (1955).

[b27] Guckenheimer J. & Oliva R. A. Chaos in the hodgkin-huxley model. SIAM J. Appl. Dyn. Syst. 1, 105–114 (2002).

[b28] Erdmann C. Investigation of threshold in neural systems. *Ph.D. Thesis*, Freie Universitität Berlin (2011).

[b29] Wechselberger M., Mitry J. & Rinzel J. Canard theory and excitability. In Nonautonomous dynamical systems in the life sciences (eds Kloeden P. E. & Pötzsche C.) 89–132 (Springer, 2013).

[b30] Kuehn C. Singularities and Canards, in Multiple Time Scale Dynamics, Applied Mathematical Sciences 191 (Springer International Publishing, Switzerland, 2015).

[b31] Izhikevich E. M. In Dynamical systems in neuroscience: The Geometry of Excitability and Bursting (The MIT press, 2007).

[b32] Izhikevich E. M. Neural excitability, spiking and bursting. Int. J. Bifurcat. Chaos 10, 1171–1266 (2000).

[b33] Mitry J., McCarthy M., Kopell N. & Wechselberger M. Excitable neurons, firing threshold manifolds and canards. J. Math. Neurosci. 3, 12 (2013). 23945278 10.1186/2190-8567-3-12 PMC3819701

[b34] Prescott S. A., De Koninck Y. & Sejnowski T. J. Biophysical basis for three distinct dynamical mechanisms of action potential initiation. PLoS Comput. Biol. 4, e1000198 (2008). 18846205 10.1371/journal.pcbi.1000198 PMC2551735

[b35] Khovanov I. A., Polovinkin A. V., Luchinsky D. G. & McClintock P. V. E. Noise-induced escape in an excitable system. Phys. Rev. E 87, 032116 (2013).

[b36] Franović I., Todorović K., Perc M., Vasović N. & Burić N. Activation process in excitable systems with multiple noise sources: One and two interacting units. Phys. Rev. E 92, 062911 (2015). 10.1103/PhysRevE.92.062911 26764778

[b37] Franović I., Todorović K., Perc M., Vasović N. & Burić N. Activation process in excitable systems with multiple noise sources: Large number of units. Phys. Rev. E 92, 062912 (2015). 10.1103/PhysRevE.92.062912 26764779

[b38] Gerstner W. & Kistler W. M. Spiking neuron models: Single neurons, populations, plasticity (Cambridge university press, 2002).

[b39] Sekerli M., Del Negro C. A., Lee R. H. & Butera R. J. Estimating action potential thresholds from neuronal time-series: new metrics and evaluation of methodologies. IEEE Trans. Biomed. Eng. 51, 1665–1672 (2004). 15376515 10.1109/TBME.2004.827531

[b40] Desroches M., Krupa M. & Rodrigues S. Inflection, canards and excitability threshold in neuronal models. J. Math. biol. 67, 989–1017 (2013). 22945512 10.1007/s00285-012-0576-z

[b41] Fenichel N. Geometric singular perturbation theory for ordinary differential equations. J. Differ. Equat. 31, 53–98(1979).

[b42] Chizhov A. V., Smirnova E. Y., Kim K. K. & Zaitsev A. V. A simple markov model of sodium channels with a dynamic threshold. J. Comput. Neurosci. 37, 181–191 (2014). 24469252 10.1007/s10827-014-0496-6

[b43] Gerstner W. & Naud R. How good are neuron models? Science 326, 379–380 (2009). 19833951 10.1126/science.1181936

[b44] FitzHugh R. Impulses and physiological states in theoretical models of nerve membrane. Biophys. J. 1, 445–466 (1961). 19431309 10.1016/s0006-3495(61)86902-6 PMC1366333

[b45] Henze D. A. & Buzsáki G. Action potential threshold of hippocampal pyramidal cells *in vivo* is increased by recent spiking activity. Neurosci. 105, 121–130 (2001). 10.1016/s0306-4522(01)00167-1 11483306

[b46] Volgushev M. . Onset dynamics of action potentials in rat neocortical neurons and identified snail neurons: quantification of the difference. PLoS ONE 3, e1962 (2008). 18398478 10.1371/journal.pone.0001962 PMC2276861

[b47] Yu Y., Shu Y. & McCormick D. A. Cortical action potential backpropagation explains spike threshold variability and rapid-onset kinetics. J. Neurosci. 28, 7260–7272 (2008). 18632930 10.1523/JNEUROSCI.1613-08.2008 PMC2664555

[b48] Colwell L. J. & Brenner M. P. Action potential initiation in the hodgkin-huxley model. PLoS Comput. Biol. 5, 1000265 (2009). 10.1371/journal.pcbi.1000265 PMC2607014 19148265

[b49] Baranauskas G., Mukovskiy A., Wolf F. & Volgushev M. The determinants of the onset dynamics of action potentials in a computational model. Neuroscience 167, 1070–1090 (2010). 20211703 10.1016/j.neuroscience.2010.02.072

[b50] Brette R. What is the most realistic single-compartment model of spike initiation? PLoS Comput. Biol. 11, e1004114 (2015). 25856629 10.1371/journal.pcbi.1004114 PMC4391789

[b51] Wang L., Wang H., Yu L. & Chen Y. Role of axonal sodium-channel band in neuronal excitability. Phys. Rev. E 84, 052901 (2011). 10.1103/PhysRevE.84.052901 22181462

