# Supplementary material for: Spike-Threshold Variability Originated from Separatrix-Crossing in Neuronal Dynamics
Source: Sci Rep. 2016 Aug 22;6:31719. doi: 10.1038/srep31719 (PMC4992847; doi:10.1038/srep31719)
Supplement: Supplementary Information [file srep31719-s1.pdf]

Spike-Threshold Variability Originated from Separatrix-Crossing in Neuronal Dynamics

Longfei Wang[1], **Hengtong Wang**[2], **Lianchun Yu**[1], **and Yong Chen**[3,4,*]

[1]Institute of Theoretical Physics, Lanzhou University, Lanzhou, Gansu 730000, China
[2]College of Physics and Information Technology, Shaanxi Normal University, Xi'an 710062, China
[3]Center of Soft Matter Physics and its Application, Beihang University, Beijing 100191, China
[4]School of Physics and Nuclear Energy Engineering, Beihang University, Beijing 100191, China
[*]ychen@buaa.edu.cn

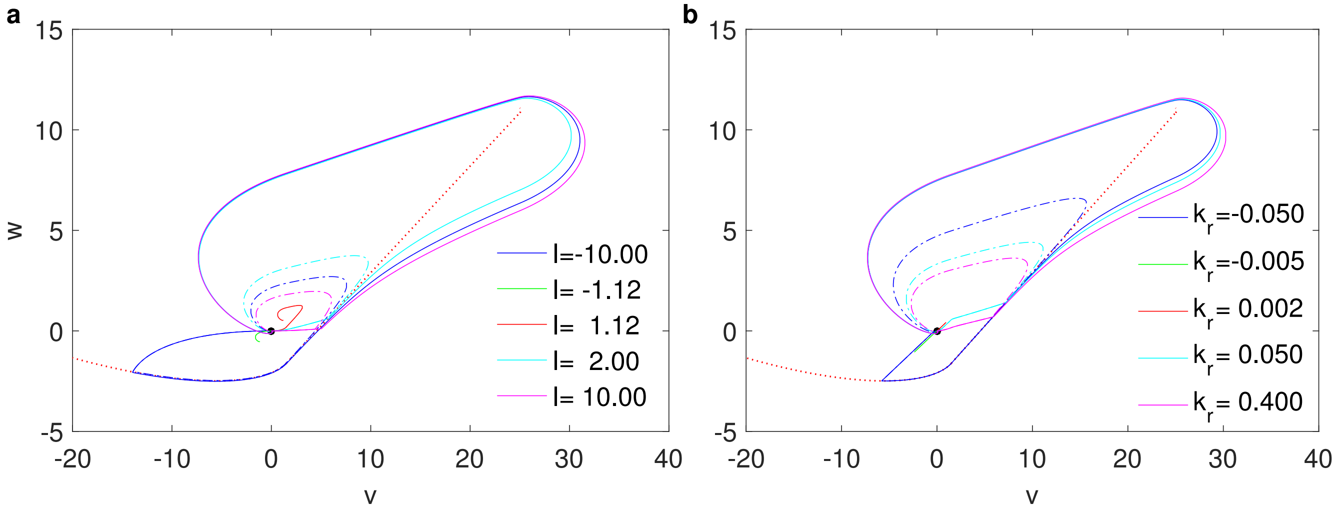

Figure S1. Threshold of step and ramp pulse determined by separatrix in 2D-PWL model. (a) Trajectories (solid lines and dash-dotted lines) of step current injections with different amplitudes to the PWL model. Whether the change after a rectangular pulse injection can induce an AP is determined by whether the state have crossed the separatrix (red dash-dotted line). In this model, a minimum threshold amplitude (I_{thr}) must be exceed to have an transient AP[1,2]. (b) Trajectories of ramp current injection with different rate to PWL model. Whether AP will generate after the ramp current also determined by separatrix-crossing mechanism. The pluses' duration of every solid line and the corresponding dash-dotted line with the same color differ 0.06.

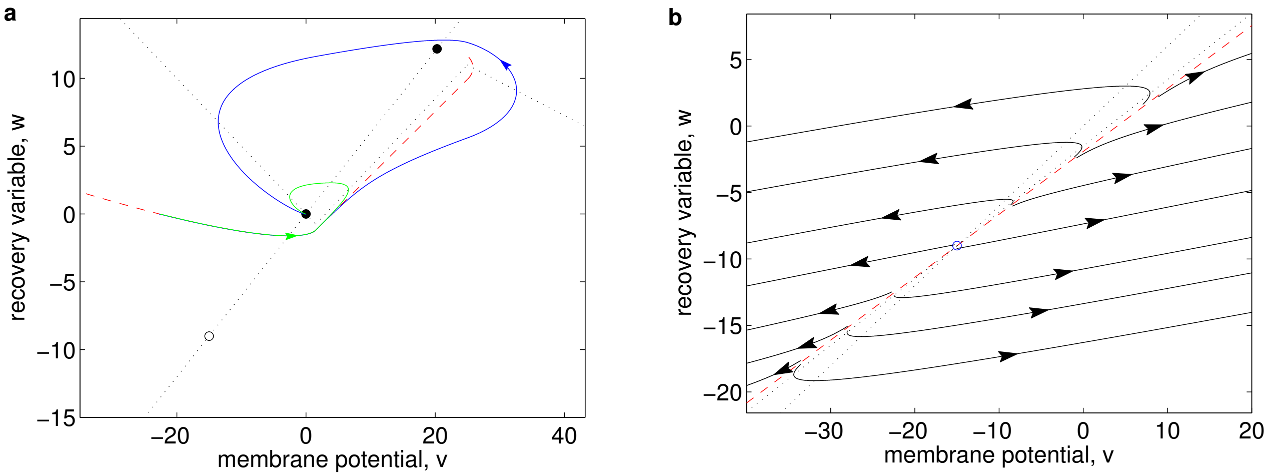

Figure S2. An unstable separatrix of unstable node determines threshold voltages in 2D-PWL model. (a) Trajectories (solid lines with arrows) show the unstable separatrix of unstable node (the open circle at the bottom) does act as the threshold set of the system (the red dashed lines). Black dotted lines are the nullclines. (b) The local trajectories of the unstable node (expand v definition domain of middle segment in (a) to the whole v-axis). The parameters are: $k_l = -0.5$, $k_m = 0.5$, $k_r = -0.25$ $k_w = 0.6$, $v_l = 1.5$, $v_r = 25.0$, $\tau_w = 10.0$, $i_e(t) = 0$.

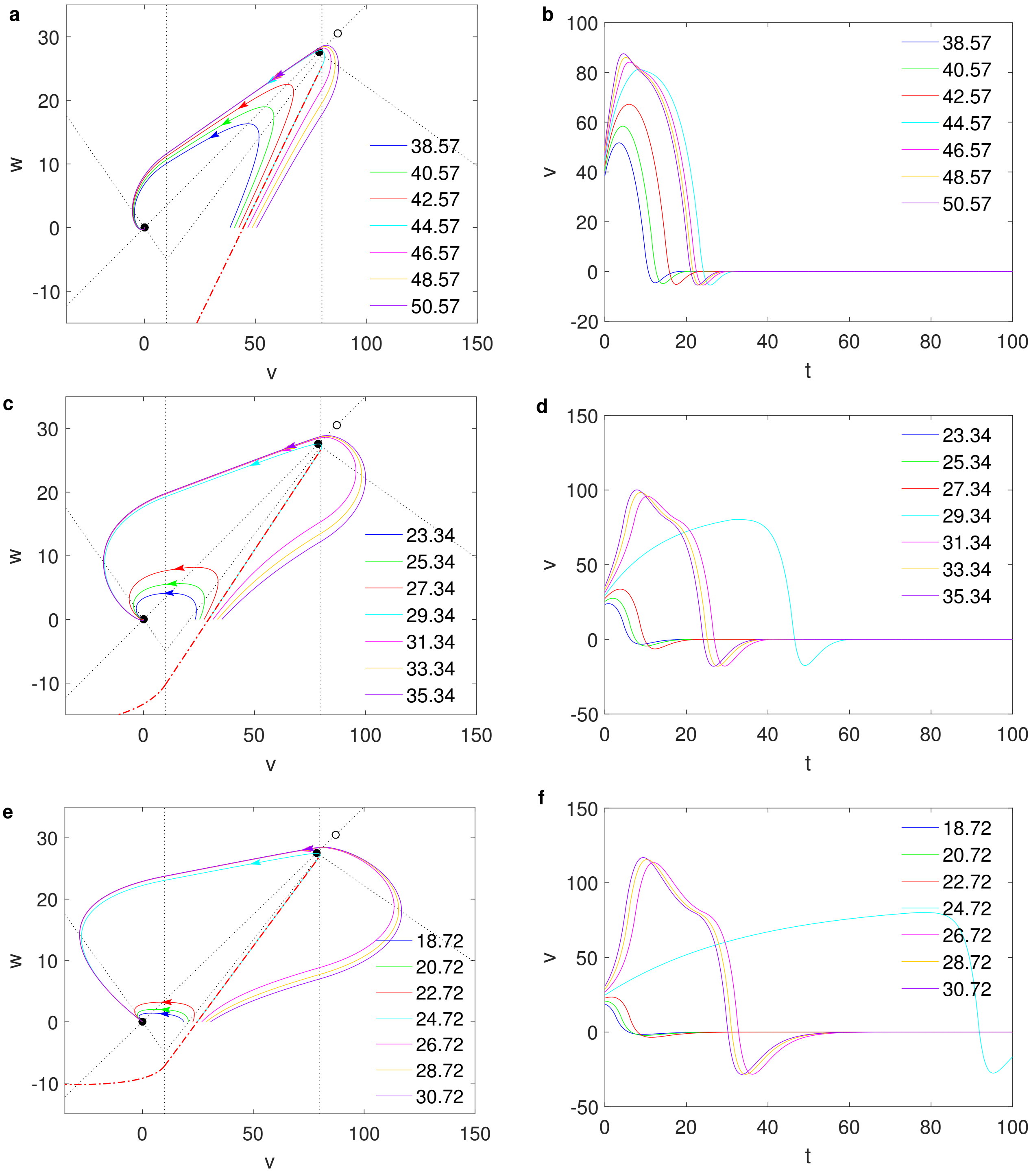

Figure S3. The time constant affect the distinctiveness of threshold voltage of PWL model. The state plane trajectories and time series of different initial voltages. The threshold phenomena become more distinct as increasing of the time constant of recovery variable w, which in subfigures are the following values: (a) & (b), $\tau_w = 2.0$; (c) & (d), $\tau_w = 5.0$; (e) & (f) ,$\tau_w = 10.0$ Other parameters are: $k_l = -0.5$, $k_m = 0.46$, $k_r = -0.25$, $k_w = 0.35$, $v_l = 10$, $v_r = 80$, $i_e(t) \equiv 0$.

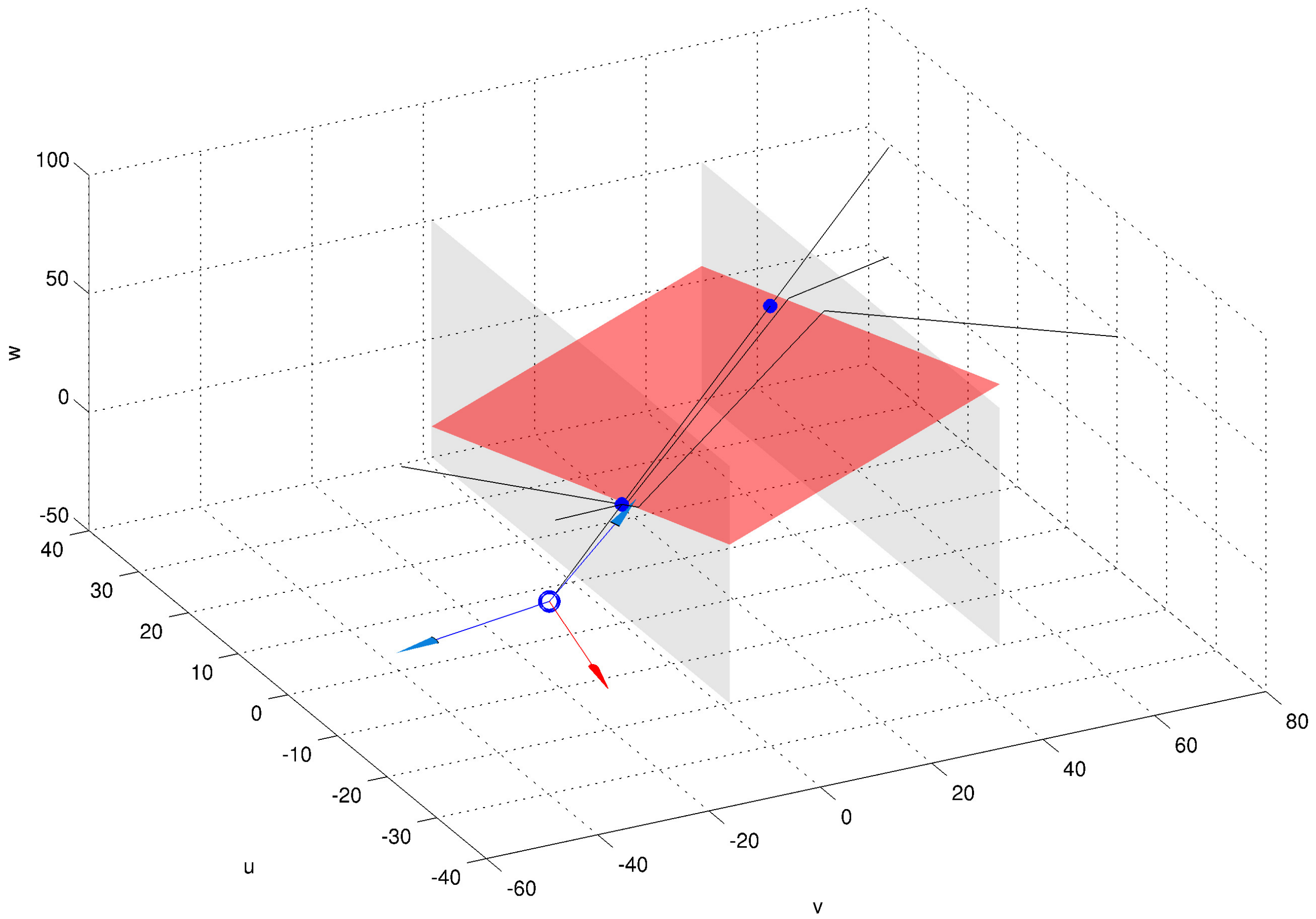

Figure S4. Dynamic analysis of 3D-PWL model. Any pair equations of our 3D-PWL model define a nullcline in state space (black solid lines). Three nullclines intersect with each other in real or by elongating at three fixed points, however, only the one representing the resting potential is actually exists, the other two are virtual, i.e. not locate in their definition zone. The sepratrix separate the 3-dimensional space into two parts which defines the firing or non-firing region in the middle region. Arrows show the characteristic vectors, the red one is the one with negative characteristic eigenvalue.

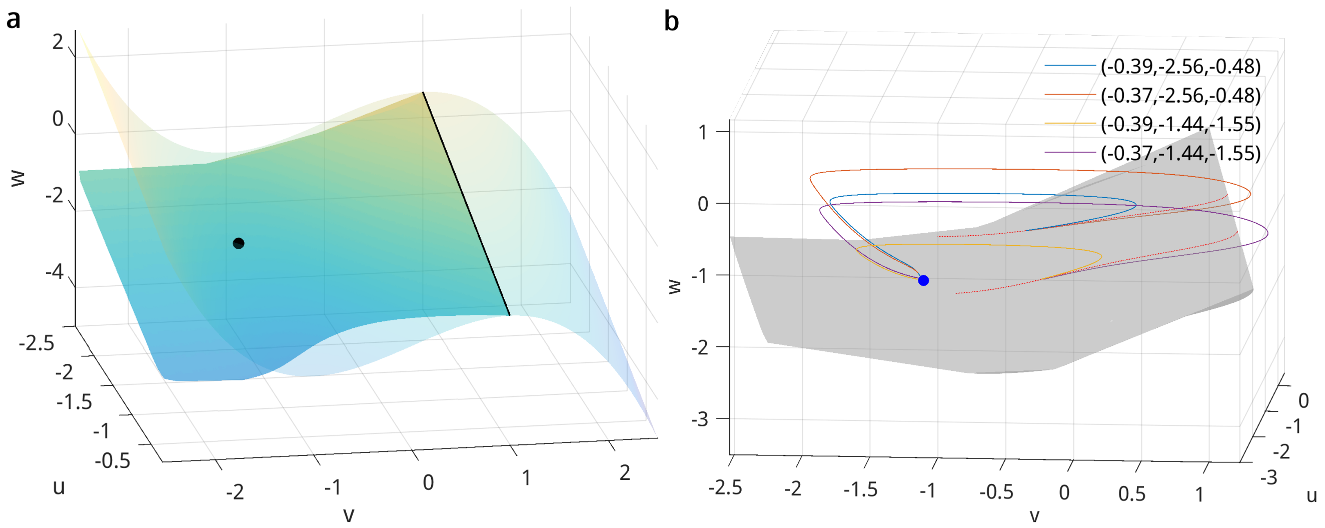

Figure S5. **Separatrix in 3D FHN model.** (a) Fixed point (black point), nullcsurface (front surface), fold curve (black line) and the separatrix (surface behind the nullcsurface). (b), The canard trajectories passing through the points of fold curve determine the threshold.

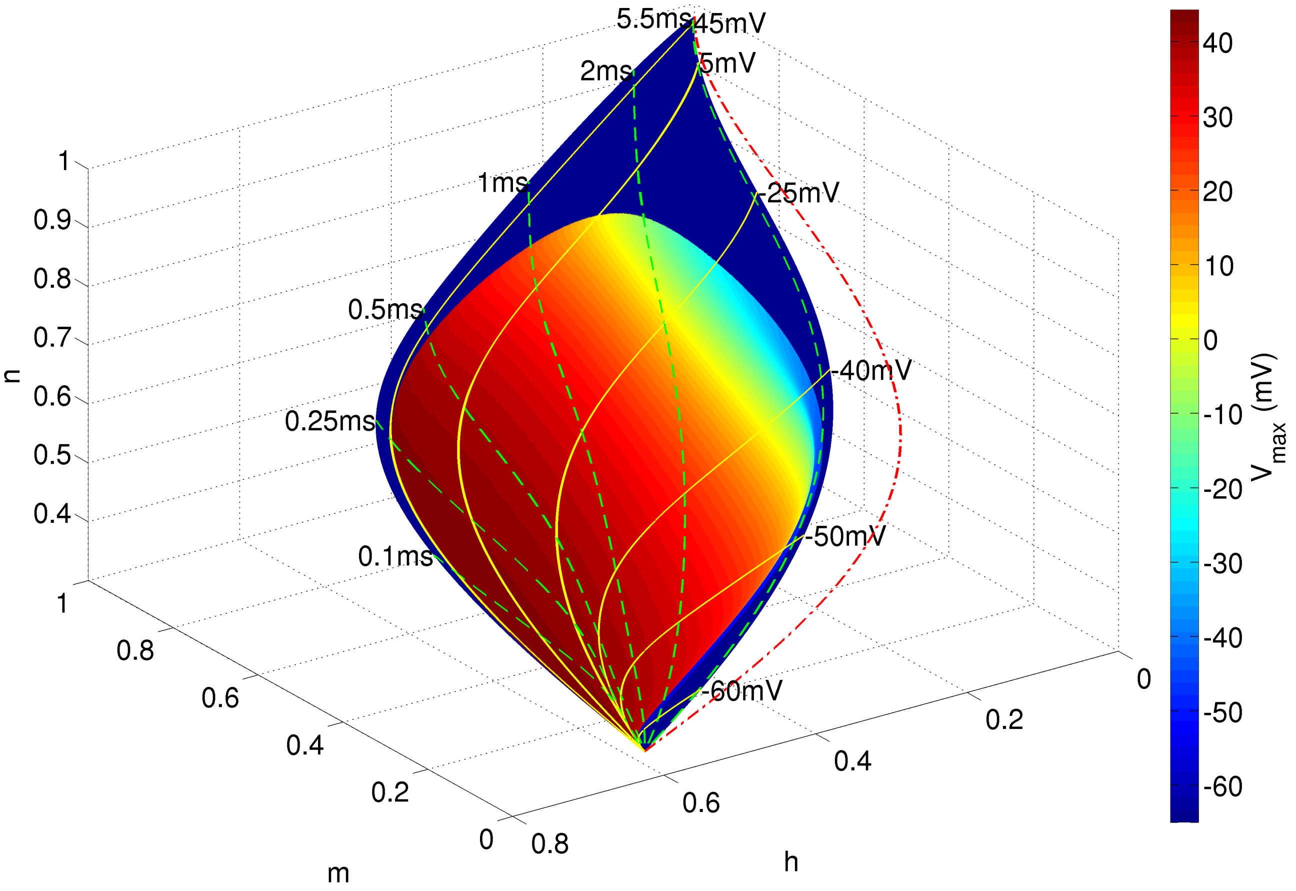

Figure S6. **Separatrix-crossing mechanism of HH model in *m*-*h*-*n* state space.** The boundary of maximum voltages after voltage clamp mapped on voltage clamp surface in *m*–*h*–*n* state space numerically show the separatrix of HH model. Colors indicate different maximum voltages after voltage clamp. Yellow solid lines show voltage clamping processes at several voltages and the green dashed lines indicate same duration of voltage clamping, they are all marked with values at the end of the lines. On the separtrix, the instantaneous threshold (θ) equal to clamping voltage V_c. In the firing zone (hot colored region), we have $V_c > \theta$ and in the blue region $V_c < \theta$ when voltage clamping is off.

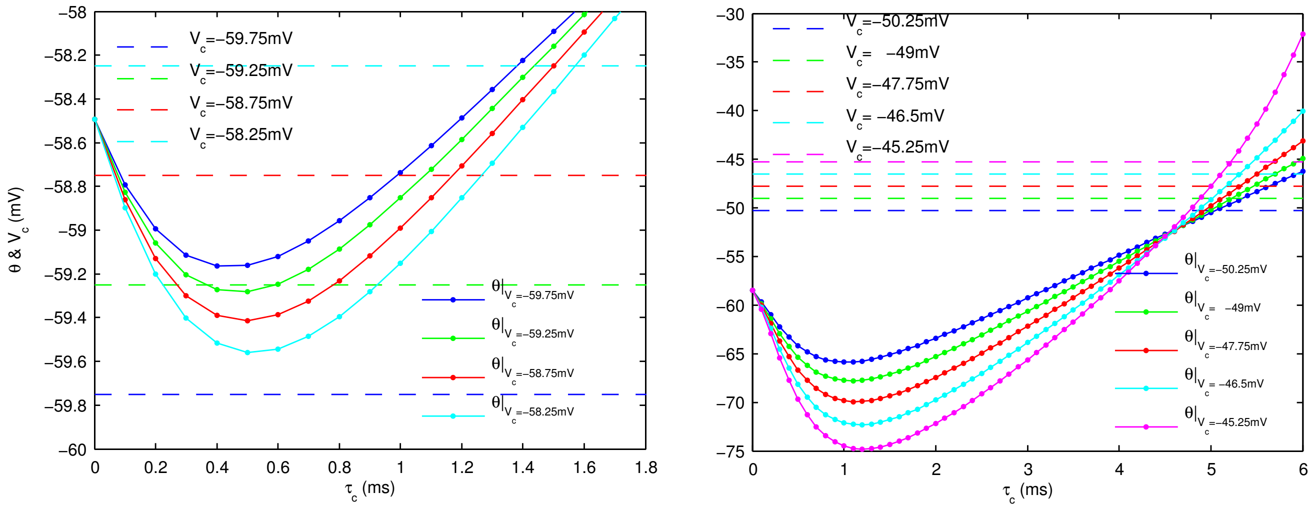

Figure S7. **Points where clamped voltage and instantaneous threshold equal (i.e. $V_c = \theta$) form threshold voltages/times line.** Left: The formation of minimum threshold voltage. Right: The formation of maximum clamping time which can induce AP. The criteria of AP is $V_{max} \geqslant -15mV$, so threshold points will form the $-15mV$ line in the contour map of maximum voltage (Figure S5).

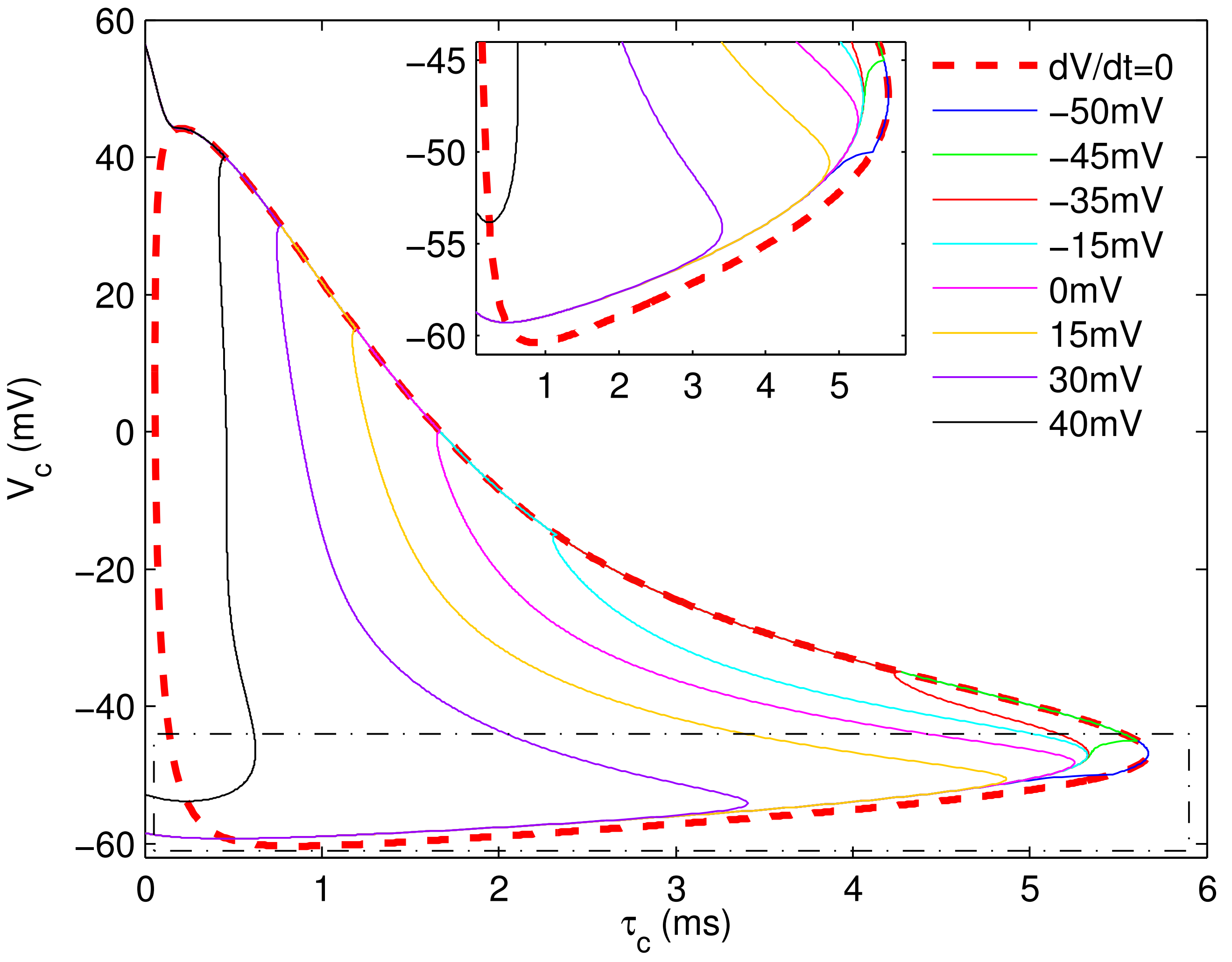

Figure S8. **Contour of maximum voltage after voltage clamp in τ_c and V_c phase space.** All contour lines consist of three segments: low voltage segment (indicating normal threshold), intermediate voltage segment and high voltage segment (overlapping with $dV/dt = 0$ at the moment the voltage clamping is off).

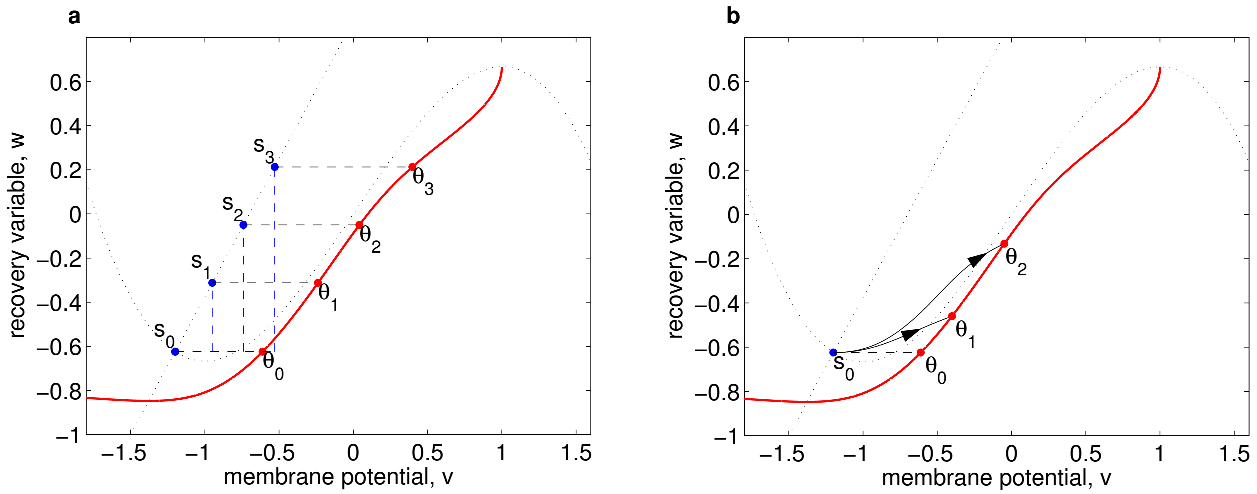

Figure S9. **The deterministic version of threshold related to membrane potential and rising rate of depolarization.** (a) Threshold for different clamped states. $\theta_i\,(i=0,1,2,3)$ are the corresponding threshold of $s_i\,(i=0,1,2,3)$ respectively. The threshold increases with the membrane potential. (b) Threshold decreases with depolarizing rate of membrane potential induced by step current. The instantaneous current $I_0\delta(t)$ horizontally shifting of resting state have the lowest threshold for depolarizing current injection. From resting state point s_0 is brought to threshold point θ_2 by step current with amplitude 0.164 ($\Delta t = 11.67575$), while the step current bring state from s_0 to θ_1 having amplitude 0.2 ($\Delta t = 4.7875$).

Note S1: Derivation of the threshold curve of 2D-PWL model

As shown in Fig. 3, v-nullcline have three segments and intersect with w-nullcline at three points (fixed points). This set of parameters makes the three fixed points, two stable spiral (focus) and a saddle, from left to right in the phase plane, respectively. The stable manifolds of saddle (separatrices) act as instantaneous threshold voltages in this PWL model. The separatrix s is a straight-line because the nullclines are linear. We assumed that the separatrix is $w = k_\theta v + b_\theta$, and we calculated the slope rate $k_\theta = 2k_w C / \left(k_m \tau_w + C - \sqrt{(k_m \tau_w + C)^2 - 4k_w \tau_w} \right)$ and the w intercept $b_\theta = \frac{k_w - k_\theta}{k_w - k_m}(i_e + b_m)$. Then we obtained threshold voltages:

$$\theta = \frac{1}{k_\theta}(w - b_\theta). \tag{S1}$$

In equation (S1), we replaced w with the transient value determined by the voltage clamping process:

$$w = k_w v + (w_0 - k_w v) \mathrm{e}^{\frac{-t}{\tau_w}}, \tag{S2}$$

then we obtained the corresponding quasi-separatrix in the clamped voltage versus clamping time (τ_c-v_c) plane:

$$\theta(\tau_c) = \frac{w_0 \mathrm{e}^{-\frac{\tau_c}{\tau_w}} - b_\theta}{(1 - \mathrm{e}^{-\frac{\tau_c}{\tau_w}})k - k_\theta}. \tag{S3}$$

The corresponding curve of $dv/dt = 0$ in the $\tau_c - v_c$ plane also has a similar form.

Note S2: Derivation of the threshold plane of 3D-PWL model

Shown in Supplementary Fig. S2, the piecewise linear function $f(v)$ divides the whole space into three parts. For the above parameters, the left part has a real equilibrium, which is a stable node and represents resting state; the middle part has a virtual equilibrium in the left part, which is a saddle with its stable manifold consisting of thresholds; and the right part has a virtual equilibrium in the middle part, which is also a stable node and functions as a recovery mechanism. In order to show the dynamical properties well, we plot the nullclines (the intersect lines of nullcplanes $dx/dt = 0\,(x = v,u,w)$) only.

The threshold set of our 3D-PWL model is a plane. See Figure 5 and S2, the plane is determined by stable (red arrow) and unstable (blue arrow) eigenvectors of a equilibrium (the blue circle). Each equation of 3D-PWL model defines a plane (three planes by the piecewise linear equation of dv/dt), whereas any satisfied pair of equations forms a nullcline in state space. These three nullclines intersect with each other at three fixed points; however, only the one that represents the resting potential is actually exists, the other two are virtual, i.e., not located in their definition zone (see Figure S2). The saddle is

$$(v_f, u_f, w_f) = v_f \cdot (1, k_u, k_w),$$

where $v_f = \frac{b_m + i_e}{k_u + k_w - k_m}$. According to dynamical system theory, we obtain the following eigenvalues and eigenvectors of the characteristic matrix:

$$\lambda_i = \frac{r_i}{\tau_u \tau_w C}, \tag{S4}$$

$$\mathbf{v}_i = \left(\frac{r_i + t_u C}{C k_w \tau_u}, \frac{r_i \tau_u (k_m \tau_w - C) + \tau_u^2 \tau_w C (k_m - k_w) - r_i^2}{k_w \tau_u^2 \tau_w}, 1 \right), \tag{S5}$$

where $r_i\,(i = 1,2,3)$ is the i-th root of the equation

$$x^3 + (C(\tau_u + \tau_w) - k_m \tau_u \tau_w) x^2 + (C + \tau_u (k_w - k_m) + \tau_w (k_u - k_m)) C \tau_u \tau_w x + (k_u + k_w - k_m) C^2 \tau_u^2 \tau_w^2 = 0. \tag{S6}$$

Using the fixed-point coordinates and two eigenvectors, the threshold plane can be written using a point-norm form equation:

$$-k_w \tau_u C (r_i + r_j + \tau_u (C - k_m \tau_w)) (v - v_f) - k_w \tau_u^2 \tau_w C (u - u_f) + (r_i (r_j + \tau_u C) + \tau_u (r_j + \tau_u (C - k_w \tau_w))) (w - w_f) = 0, \tag{S7}$$

where $i \neq j$. So the explicit equation of the threshold can be written as

$$\theta = -au - bw + (1 + a k_u + b k_w) v_f, \tag{S8}$$

where $a = \frac{\tau_u \tau_w}{r_i + r_j + \tau_u (C - k_m \tau_w)}$ and $b = -\frac{r_i (r_j + \tau_u C) + \tau_u (r) h + \tau_u (C - k_w \tau_w))}{k_w \tau_u C \left(r_i + r_j \tau_u (C - k_m \tau_w) \right)}$.

References

1. Izhikevich, E. M. Dynamical systems in neuroscience. (The MIT press, 2007).
2. Wechselberger, M., Mitry, J. & Rinzel, J. Canard theory and excitability. In *Nonautonomous dynamical systems in the life sciences*, 89-132 (Springer, 2013).
